# Oxygen atom transfer with organofunctionalized polyoxovanadium clusters: O-atom vacancy formation with tertiary phosphanes and deoxygenation of styrene oxide†
†Electronic supplementary information (ESI) available: ^1^H NMR, ESI-MS (+ve), UV-vis, and IR spectroscopic data for all complexes, crystallographic parameters and BVS calculations of complexes **2-OPMe_3_**, **2-OPMe_2_Ph**, **2-OPMePh_2_**, **2-OPPh_3_**, and **4-OPPh_3_**, attempted synthesis of **4-OPMePh_2_**, **4-OPPh_3_**, and propoxide derivatives, ^1^H NMR analysis of styrene oxide OAT reactions with **2-OPMe_3_** and **4-OPMe_3_**. For ESI and crystallographic data (CCDC1914250–1914254) in CIF or other electronic format see DOI: 10.1039/c9sc02882j


**DOI:** 10.1039/c9sc02882j

**Published:** 2019-07-15

**Authors:** Brittney E. Petel, Rachel L. Meyer, William W. Brennessel, Ellen M. Matson

**Affiliations:** a Department of Chemistry , University of Rochester , Rochester , New York 14627 , USA . Email: matson@chem.rochester.edu

## Abstract

We report a rare example of oxygen atom transfer (OAT) from a polyoxometalate cluster to a series of tertiary phosphanes. Addition of PR_3_ (PR_3_ = PMe_3_, PMe_2_Ph, PMePh_2_, PPh_3_) to a neutral methoxide-bridged polyoxovanadate-alkoxide (POV-alkoxide) cluster, [V_6_O_7_(OMe)_12_]^0^, results in isolation of a reduced structure with phosphine oxide datively coordinated to a site-differentiated V^III^ ion. A positive correlation between the steric and electronic properties of the phosphane and the reaction rate was observed. Further investigation of the steric influence of the alkoxy-bridged clusters on OAT was probed through the use of POV clusters with bridging alkoxide ligands of varying chain length ([V_6_O_7_(OR′)_12_]; R′ = Et, ^*n*^Pr). These investigations expose that steric hinderance of the vanadyl moieties has significant influence on the rate of OAT. Finally, we report the reactivity of the reduced POV-alkoxide clusters with styrene oxide, resulting in the deoxygenation of the substrate to generate styrene. This result is the *first* example of epoxide deoxygenation using homometallic polyoxometalate clusters, demonstrating the potential for mono-vacant Lindqvist clusters to catalyze the removal of oxygen atoms from organic substrates.

## Abstract 2

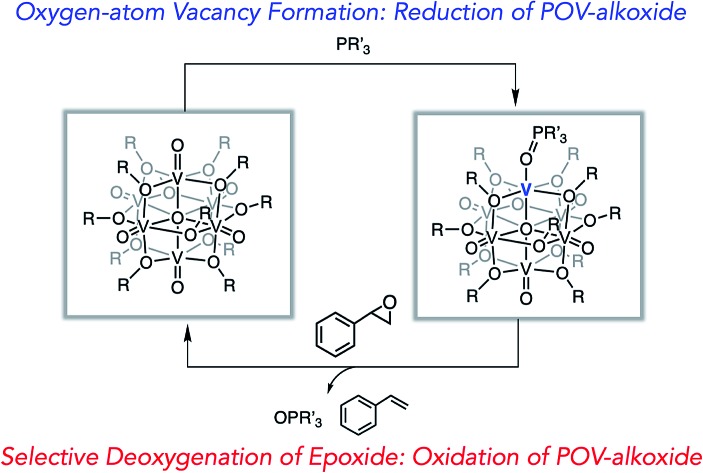
We report a rare example of oxygen atom transfer (OAT) from a polyoxometalate cluster to a series of tertiary phosphanes followed by OAT from styrene oxide to the reduced scaffold, resulting in the formation of styrene.

## Introduction

Oxygen atom transfer (OAT) is a key step in catalytic transformations with relevance to the activation of small molecules and organic substrates involving metal oxide systems. Specifically, heterogeneous reducible metal oxide materials have been demonstrated to facilitate OAT reactions *via* the Mars–van Krevelen mechanism.^1^–^8^ In chemical reactions of this type, the metal oxide surface is activated by a substrate, resulting in the formation of an oxygen-atom defect (“vacancy”). These reactive sites are thought to be key in the activation of chemically inert, gaseous substrates, including CO_2_ and NO, serving as O-atom *acceptors* from these substrates in small molecule activation.^3^–^7^

Given the prevalence of metal oxide materials capable of mediating OAT reactions, chemists have long sought well-defined, molecular species that can serve as structural and functional models of these heterogeneous catalysts. Polyoxometalates (POMs), are ideal molecular entities for modelling OAT reactivity with metal oxide systems due to their modularity, stability, and ability to undergo reversible multi-electron redox processes.^9^–^15^ The inherent physicochemical properties of these assemblies are credited to their molecular composition, containing early transition metal ions in their highest oxidation states (M = Mo^VI^, W^VI^, V^V^, Nb^V^), bridged by oxide (O^2–^) ions. Previous work focusing on the reactivity of POMs have revealed that these clusters are effective catalysts for a variety of oxidative chemical transformations, ranging from C–H oxidation^16^–^19^ and C–E (E = C, Sn) bond cleavage,^20^–^22^ to OAT to arenes, alkylarenes, and sulphides.^23^–^25^

These seminal examples of oxidation catalysis mediated by POMs have sparked extensive research in OAT chemistry of these clusters over the past thirty years. In particular, the reactivity of the phosphovanadomolybdate clusters of the α-Keggin framework, [H_3+*x*_PV_*x*_Mo_12–*x*_O_40_] (*x* = 0, 1, 2), popularized by Neumann^18^,^19^,^26^–^28^ and others,^25^,^29^ has dominated this chemical space, illustrating novel OAT chemistry for soluble, metal oxide assemblies. The authors have proposed that these clusters mediate OAT through a Mars–van Krevelen mechanism, in analogy to heterogeneous bulk metal oxide systems.^16^,^17^ Theoretical investigations, supported by spectroscopic analysis, have revealed that OAT occurs *via* an initial one-electron reduction of the polyoxoanion by the substrate, resulting in the formation of a reactive ion pair. In a subsequent mechanistic step requiring water, the oxidation of said substrate occurs.^16^,^21^,^30^–^33^ Density functional theory supports the transient formation of a coordinatively unsaturated vanadium ion at the surface of the cluster that rapidly reacts with a terminal oxidant to restore the vacant site. Given the significance of the proposed reduced species in these molecular transformations, extensive efforts have focused on the isolation and characterization of oxygen-deficient POMs. However, to date, examples of well-defined polyoxoanions containing a coordinatively unsaturated, reduced ion, generated *via* reduction of the parent metal oxide cluster, remain scarce.^29^,^34^–^37^

Here, we report a rare example of OAT from homometallic polyoxovanadate-alkoxide (POV-alkoxide) clusters to phosphane substrates in nonaqueous media. These hydrophobic, organofunctionalized metal oxide assemblies are completely insoluble/immiscible with water, thus eliminating the possibility of oxygen-atom abstraction occurring *via* water/proton assisted pathways. Furthermore, the bridging alkoxide ligands isolate oxygen atom vacancy formation to terminal oxide positions, preventing formation of coordinatively unsaturated species with defects at thermodynamically preferred bridging sites. Using paramagnetic ^1^H NMR spectroscopy, electrospray ionization mass spectrometry (ESI-MS), and X-ray crystallography, we unambiguously confirm formation of a reduced polyoxovanadium cluster *via* OAT to a phosphane. The resulting site-differentiated vanadium(iii) centre of the Lindqvist core is coordinated to a dative phosphine oxide ligand, as confirmed by crystallography and IR spectroscopy. Subsequent investigations surrounding structure–activity relationships of both the phosphane and the POV-alkoxide cluster are undertaken, revealing the importance of steric accessibility for the direct transfer of an oxygen atom from the cluster to the substrate. This work marks the first example of such a study, unveiling not only new routes for the formation of reactive POV-alkoxide architectures, but also considerations in system design for efficient OAT.

Additionally, we present an extension of the reactivity of our reduced POV-alkoxide scaffolds, summarizing their utility in the multielectron deoxygenation of styrene oxide. While POMs are well-established for the mediation of the chemical reverse of this transformation (oxidation of alkenes),^25^,^29^,^38^–^40^ there have been limited reports of the reduction of epoxides as mediated by reduced metal ions supported by a metal-oxide framework.^41^ This distinct example of the deoxygenation of an oxidized organic substrate by oxygen deficient clusters completes a water-independent, stoichiometric OAT cycle, establishing new reactivity for homometallic POM assemblies.

## Results and discussion

### O-atom vacancy formation with tertiary phosphanes: reduction of [V_6_O_7_(OMe)_12_] with PMe_3_

**Scheme 1:**
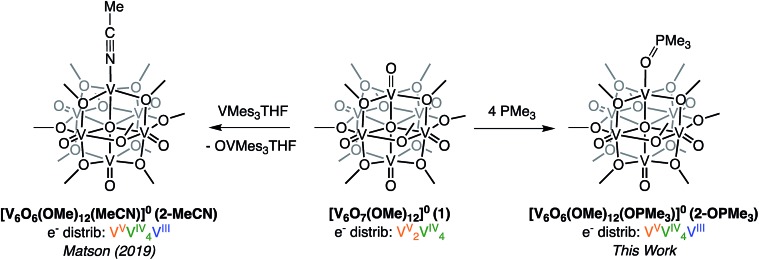
Previously reported synthesis of the mono-vacant neutral cluster, **2-MeCN**, *via* addition of VMes_3_(THF) addition (left)^35^ and synthesis of [V_6_O_6_(OMe)_12_OPMe_3_] (**2-OPMe_3_**) *via* OAT to PMe_3_ reported in this work (right).

Recently, we disclosed O-atom vacancy generation at the surface of a neutral POV-alkoxide scaffold, demonstrating that the addition of a single equivalent of V(Mes)_3_(THF) (Mes = 2,4,6-trimethylphenyl) to the parent cluster, [V_6_O_7_(OMe)_12_]^0^ (**1**), results in OAT from the POV-alkoxide to the reductant (Scheme 1, left).^34^ The product mixture included both the mono-vacant complex [V_6_O_6_(OMe)_12_(MeCN)]^0^ (**2-MeCN**), and the di-vacant cluster [V_6_O_5_(OMe)_12_(MeCN)_2_]^0^, whose similar solubilities made separation challenging. The “over-reduction” of **1** prompted a search for improved methods for selective generation of a neutral species containing a single O-atom defect-site.

Trivalent alkyl- or aryl-phosphanes (PR_3_) constitute a class of oxygen atom acceptors that have been demonstrated to efficiently remove O-atoms from a variety of inorganic metal–oxo complexes.^42^–^49^ As such, we began our efforts toward probing OAT by adding trimethylphosphine (PMe_3_) to complex **1** (Scheme 1, right). Analysis of the crude reaction mixture resulting from addition of 1 equiv. of PMe_3_ to complex **1** at room temperature (∼21 °C) *via*^1^H NMR spectroscopy showed, after 24 hours, small amounts of formation of a new product, with three paramagnetic resonances slightly shifted from those of the previously reported, neutral mono-vacant cluster, **2-MeCN** (Fig. S1†).^35^ Optimization of reaction conditions revealed that addition of 4 equiv. PMe_3_ to **1** at 70 °C results in the complete consumption of the parent cluster after 19 hours (Fig. S1 and S2†). ESI-MS (+ve) of the resulting species contained a single prominent signal, corresponding to the molecular mass of the parent ion plus the phosphane substrate (Fig. S3,† *m*/*z* = 866). This result, coupled with the increased number of signals and slight shift in ^1^H NMR resonances, supports that the product formed is the trimethylphosphine oxide (OPMe_3_) adduct of a POV-alkoxide cluster containing a single oxygen-atom vacancy, [V_6_O_6_(OMe)_12_(OPMe_3_)]^0^ (**2-OPMe_3_**).

**Fig. 1:**
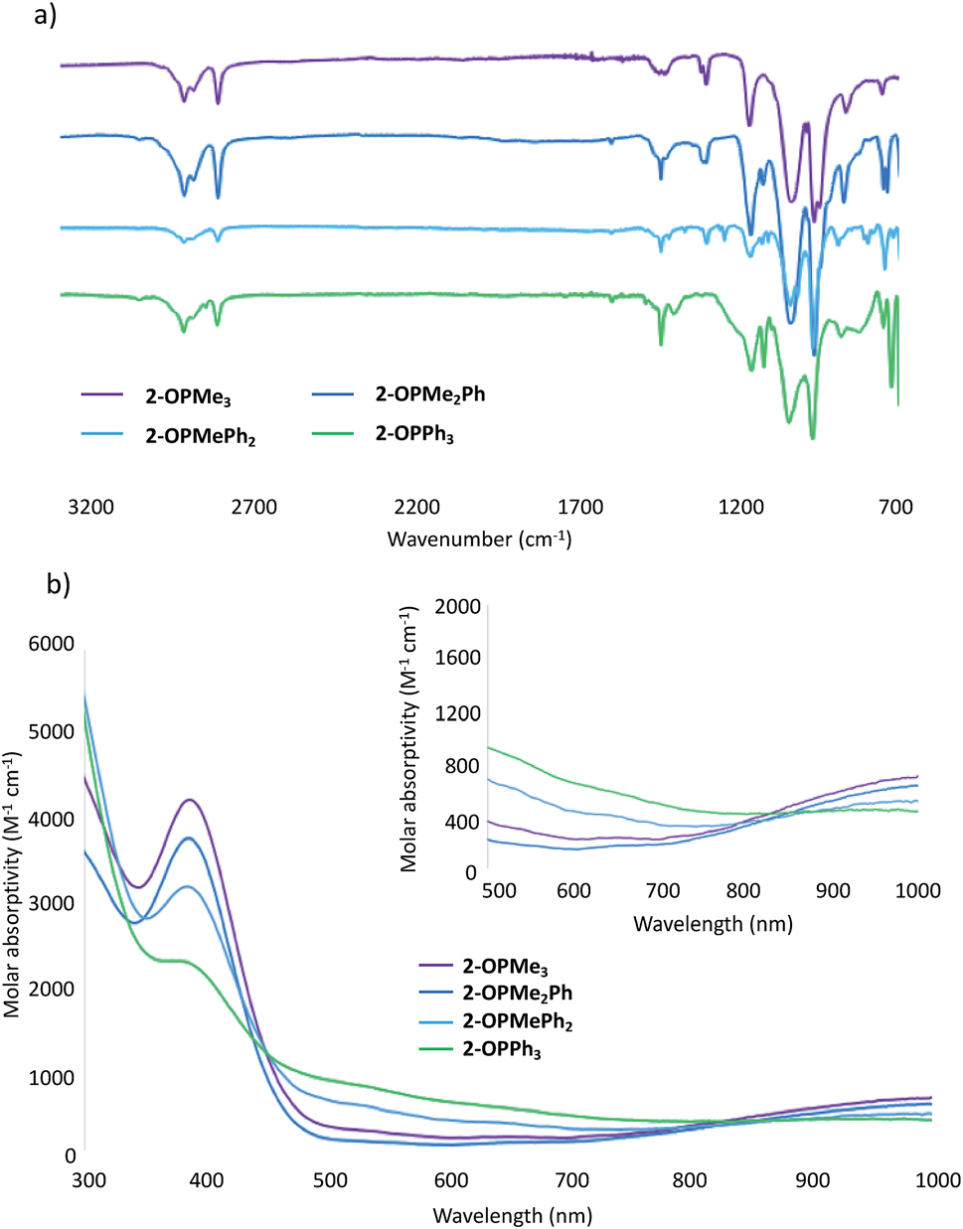
(a) Infrared spectra of complexes **2-OPMe_3_**, **2-OPMe_2_Ph**, **2-OPMePh_2_**, and **2-OPPh_3_**, see Table S7† for bond vibrations; (b) electronic absorption spectra of complexes **2-OPMe_3_**, **2-OPMe_2_Ph**, **2-OPMePh_2_**, and **2-OPPh_3_** collected in acetonitrile at 21 °C.

Further support for the generation of the reduced, phosphine oxide adduct of the cluster is observed in the infrared (IR) and electronic absorption spectra of the product (Fig. 1).^14^,^15^ In the IR spectra of **2-OPMe_3_**, two strong, broad absorption bands centred at 1032 cm^–1^ and 962 cm^–1^ are observed, corresponding to *ν*(O_b_–CH_3_) (O_b_ = bridging oxo) and *ν*(V=O_t_) (O_t_ = terminal oxo), respectively (Fig. 1a). These features resemble those reported for **2-MeCN** (*ν*(O_b_–CH_3_) 1030 cm^–1^; *ν*(V=O_t_) 968 cm^–1^), consistent with the formation of the reduced species (Fig. S4†).^35^ An additional absorbance is observed at 1163 cm^–1^, assigned to *ν*(O=P) for the bound OPMe_3_ ligand (free O=PMe_3_; 1170 cm^–1^).^50^ The similarities in energy between the *ν*(O=P) of bound and dissociated OPMe_3_ suggests *complete* OAT from the cluster to the substrate.

The electronic absorption spectrum of **2-OPMe_3_** features two pronounced intervalence charge transfer (IVCT) bands, located at 390 nm (*ε* = 3766 M^–1^ cm^–1^) and 1000 nm (*ε* = 655 M^–1^ cm^–1^) (Fig. 1b). These features are diagnostic of a mixed valent (V^IV^/V^V^) Lindqvist core.^14^,^15^ Comparison of the intensities of these absorptions in the starting material (**1**) and product (**2-OPMe_3_**) reveals a two-fold decrease in molar absorptivity (Fig. S5†). This result further supports reduction of the hexavanadate core upon OAT, as the change in oxidation state distribution from VV2VIV4 to V^V^VIV4V^III^ corresponds to a two-fold decrease in probability of IVCT occurrence between V^IV^ and V^V^ ions in the product, **2-OPMe_3_**.

**Fig. 2:**
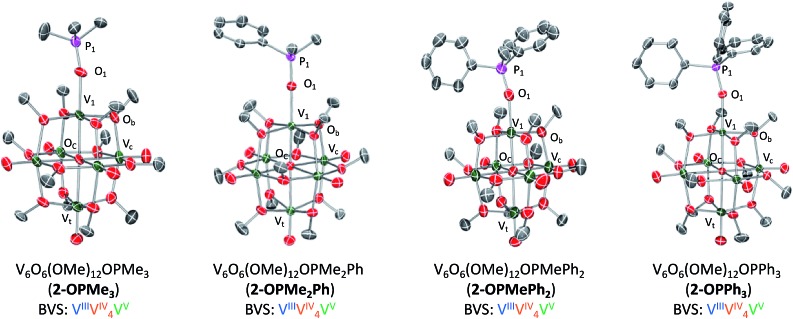
Molecular structures of **2-OPMe_3_**, **2-OPMe_2_Ph**, **2-OPMePh_2_**, and **2-OPPh_3_**. **2-OPMe_2_Ph**, **2-OPMePh_2_**, and **2-OPPh_3_** shown with 50% probability ellipsoids, **2-OPMe_3_** shown with 20% probability ellipsoids due to disorder over two positions (see Fig. S6† for more information). Hydrogen atoms removed for clarity.

Crystals suitable for structural analysis were grown from slow diffusion of pentane into a concentrated solution of **2-OPMe_3_** in tetrahydrofuran. Resolution of crystallographic data revealed the expected product; a hexavanadate core with a trimethylphosphine oxide ligand datively coordinated to the site-differentiated, reduced vanadium ion (Fig. 2 and Table 1; for full structural parameters and BVS calculations, see Tables S1 and S2†). The asymmetric unit of the crystal contained two independent molecules of **2-OPMe_3_**, with one of the molecules being highly disordered over two positions (Fig. S6†). Bond metrics of **2-OPMe_3_** were therefore obtained from the molecule in the asymmetric unit which exhibited no disorder. Complex **2-OPMe_3_** contains a lengthened V1–O1 bond in comparison to the parent POV-alkoxide cluster (**2-OPMe_3_**: 2.026(5) Å; **1** (avg): 1.589 Å, Table S3†).^14^ Additionally, the short P=O bond distance in **2-OPMe_3_** (1.467(6) Å) is comparable to the theoretically determined P=O bond length in free trimethylphosphine oxide (Me_3_P=O, 1.48 Å).^51^

**Table 1:** Selected bond lengths and angles for complexes **2-OPMe_3_**, **2-OPMe_2_Ph**, **2-OPMe_2_Ph**, and **2-OPPh_3_**. For full bond metrics and structural parameters, see ESI file

Bond	**1**	**2-OPMe_3_**	**2-OPMe_2_Ph**	**2-OPMePh_2_**	**2-OPPh_3_**
V1–O1	—	2.062(5) Å	2.0403(19) Å	2.052(14) Å	2.049(3) Å
P1–O1	—	1.467(6) Å	1.5032(19) Å	1.487(15) Å	1.506(3) Å
V1–O_c_	—	2.120(5) Å	2.0982(17) Å	1.981(13) Å	2.117(3) Å
V1–O_b_–V_*n*_ (avg)	—	105.1°	105.3°	104.2°	104.7°
V=O_t_ (avg)	1.60 Å	1.591 Å	1.600 Å	1.600 Å	1.595 Å
V–O_c_ (avg)	2.25 Å	2.314 Å	2.3245 Å	2.318 Å	2.317 Å

The lengthened V1–O1 bond and similarity in P=O bond distances between free and bound OPMe_3_ collectively provide support for a purely dative interaction between a reduced, vanadium(iii) centre and a phosphine oxide ligand. These bond metrics refute the alternative product of one electron transfer, namely the ion-paired species, [Me_3_P˙^+^···V_6_O_7_(OCH_3_)_12_].^21^,^30^,^31^ Further support for complete OAT can be garnered from examination of V1–O_c_ bond distances. Previously we have reported that reduced vanadium ions within oxygen-deficient POV-alkoxide clusters, [V_6_O_5_(OMe)_12_(MeCN)_2_]^35^ and [V_6_O_6_(OMe)_12_OTf]^34^ (OTf = trifluoromethylsulfonate), possess a shortened V1–O_c_ bond (O_c_ = central oxygen atom, 2.0173 (avg) Å, 2.08(4) Å, respectively) and contracted V1–O_b_–V_*n*_ bond angles (O_b_ = bridging oxygen atom, avg ∼105°, Table S3†). Similar deviations in bond lengths and angles are observed in the X-ray structure of **2-OPMe_3_**, with a V1–O_c_ bond length of 2.120(5) Å and V1–O_b_–V_*n*_ bond angles averaging ∼105°. Bond valence sum (BVS) calculations revealed an oxidation state distribution of vanadium ions of V^V^VIV4V^III^, consistent with that reported for **2-MeCN** (Table S2†).^35^

Direct OAT between a metal-oxide cluster and phosphane is exceedingly rare.^49^,^52^ An original report by Kawafurane described OAT from the Keggin ion [PMo_12_O_40_]^3–^, resulting in the formation of a series of reduced clusters with O-atom vacancies, [PMo_12_O_40–*x*_]^3–^ (*x* = 1, 2, 3).^53^ However, Mattes^54^ and Proust^55^ have separately revisited this work, clarifying that OAT to triphenylphosphine actually occurs *via* reductive quenching of a transiently formed PPh_3_˙^+^ species by water, with no evidence for the formation of an oxygen-atom vacancy at the surface of the cluster. To date only one example of direct OAT from a polyoxoanion to a tertiary phosphane has been reported. In the case of their chromium-functionalized tungstate ion, [XW_11_O_39_Cr^V^O]^*n*–^ (X = P, Si; *n* = 4, 5, respectively), Hill and coworkers cite reduction of the Cr^V^ heteroion to Cr^III^ upon addition of triphenylphosphine.^29^ While this work is of seminal importance, the OAT reactivity of this cluster is localized to the heteroion embedded within the polyoxotungstate structure. Indeed, heterometals in these types of clusters are well-established to be significantly decoupled from the electronic structure of the whole, with redox events localized to the heteroatom.^11^,^56^–^58^ This fact differentiates our work from that reported previously, as we present the OAT reactivity of a *homometallic* POM to an organophosphane.

### Tuning oxygen atom transfer through variation of PR_3_

**Scheme 2:**
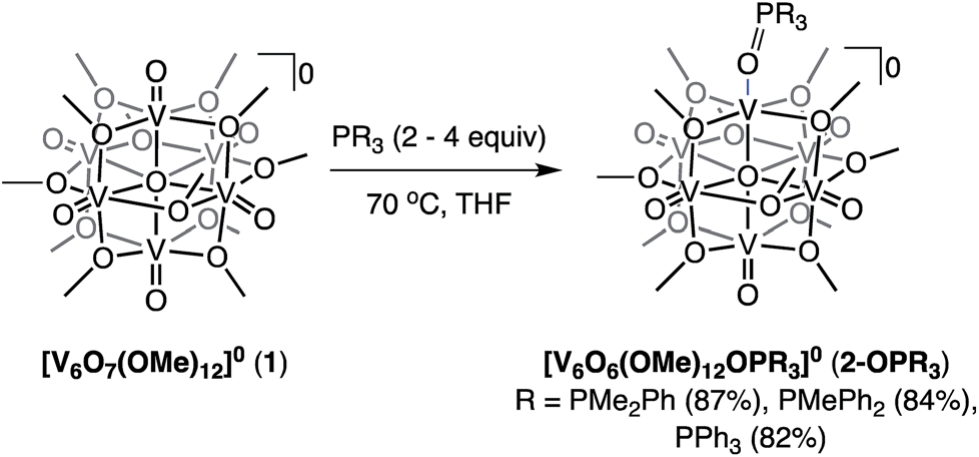
Synthesis of complexes **2-OPMe_2_Ph**, **2-OPMePh_2_**, and **2-OPPh_3_**.

Given the ability to tune the reactivity of phosphanes by altering their steric or electronic properties, we shifted our focus to the implications of phosphane identity in OAT.^59^ Addition of excess phosphane (2–4 equiv. of PMe_2_Ph, PMePh_2_, or PPh_3_) to **1** in THF at 70 °C resulted in the formation of a series of alkyl- and aryl-phosphine oxide-bound clusters in good yield (**2-OPMe_2_Ph**; 83%, **2-OPMePh_2_**; 87%, and **2-OPPh_3_**; 76%), as indicated by ^1^H NMR spectroscopy and ESI-MS (Scheme 2 and Fig. S7–S10†). The formation of the desired phosphine-oxide adducts was confirmed *via* X-ray crystallography of complexes **2-OPMe_2_Ph**, **2-OPMePh_2_** and **2-OPPh_3_** (Fig. 2 and Table 1; for full structural parameters and BVS calculations, see Tables S4–S6†). BVS calculations, supported by subsequent analytical investigations (IR and UV-vis spectroscopies), established retention of the Lindqvist core and indicate similar oxidation state distributions of vanadium ions in the reduced clusters (Fig. 1a, b and Table S7†).

While nominal changes in the molecular and electronic structures of the reduced phosphine-oxide adducts of the POV-methoxide clusters were observed when altering the basicity or cone angle of the tertiary phosphine, large variations in reaction times were obtained. Reaction optimization revealed that as phosphine nucleophilicity decreased, and cone angle increased, longer reaction times were required for complete reduction of the parent cluster, **1**. These results follow kinetic trends reported previously for OAT between PR_3_ and various metal–oxo complexes (Table S8†).^42^,^60^

The only exception to the aforementioned trend is noted in comparing the qualitative reaction rate of OAT in the case of PMe_3_ and PMe_2_Ph. Despite its smaller cone angle and increased nucleophilicity, reduction of **1** by PMe_3_ took 19 hours, whereas consumption of the starting material was observed after only 7 hours in the case of PMe_2_Ph. Furthermore, synthetic optimization for **2-OPMe_2_Ph** revealed that modelling reaction conditions after **2-OPMe_3_**, through use of 4 equiv. of reductant, resulted in a mixture of the desired product and the over-reduced, (OPMe_2_Ph)_2_-bound molecule, [V_6_O_5_(OMe)_12_(OPMe_2_Ph)_2_]^0^, confirmed by ^1^H NMR spectroscopy and ESI-MS (+ve) after 2 hours (Fig. S11 and S12†). Selective formation of **2-OPMe_2_Ph** required a decrease in reductant equivalents and time (2 equiv., 7 hours). We attribute the discrepancy in reaction rate to the volatility of the PMe_3_ (b.p. = 38 °C) *versus* that of PMe_2_Ph (b.p. = 75 °C); at the requisite elevated reaction temperatures, PMe_3_ spends a majority of its time in the headspace of the pressure vessel, rendering this reaction heterogeneous.

### Oxygen atom transfer on extended alkoxy-bridged POV-alkoxide clusters

In an effort to decouple the steric and electronic influences of OAT from the POV-alkoxide cluster to tertiary phosphanes, we extended our investigations to Lindqvist architectures with longer bridging alkoxide chains. Our group and others have previously reported that increasing the chain length of the bridging alkoxides has no effect on the electronic structure of the Lindqvist core.^15^,^61^,^62^ Thus, increasing the length of the bridging ligands surrounding the vanadyl moieties would serve to deconvolute the electronic and steric influences on OAT.

**Scheme 3:**
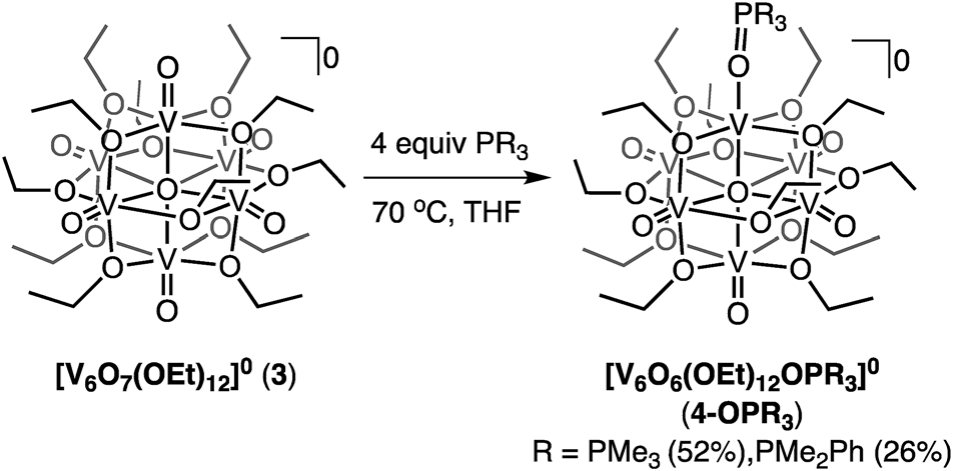
Synthesis of complexes **4-OPMe_3_** and **4-OPMe_2_Ph**.

We initiated these efforts by investigating the reactivity of PMe_3_ with the ethoxide-bridged Lindqvist cluster, [V_6_O_7_(OEt)_12_]^0^ (**3**). Addition of 4 equiv. of PMe_3_ to the POV-ethoxide cluster resulted in a gradual colour change from green to brown, after 4 days at 70 °C (Scheme 3). The increased steric bulk at the vanadyl moieties of the POV-ethoxide cluster results in a 4-fold increase in reaction time required for complete starting material consumption as compared to its POV-methoxide analogue. Spectroscopic characterization of the reduced product was consistent with formation of [V_6_O_6_(OEt)_12_OPMe_3_]^0^ (**4-OPMe_3_**; Fig. S13–S15 and Table S9†).

Isolation of **4-OPMe_3_** prompted further investigation of the reactivity of **3** with the previously described family of mixed alkyl- and aryl-phosphanes (PMe_*n*_Ph_3–*n*_; *n* = 0–3). Thus, we next explored the reactivity of **3** with PMe_2_Ph (Scheme 3). After four days at 70 °C the expected colour change from green to brown was observed. Analysis of the crude product by ^1^H NMR spectroscopy in MeCN-d_3_ revealed the expected paramagnetic resonances of the reduced product, however each possessing a single shoulder (Fig. S16†). This observation suggests the presence of multiple species in solution. Given the increased bulk of both the phosphane and the bridging moieties on the POV-alkoxide cluster, we hypothesized that steric hinderance could drive partial dissociation of the phosphine oxide ligand, OPMe_2_Ph, in coordinating solvents (MeCN-d_3_). This would result in a mixture of products, with one set of paramagnetic resonances corresponding to the expected product ([V_6_O_6_(OEt)_12_OPMe_2_Ph]^0^, **4-OPMe_2_Ph**), and the other belonging to a cluster containing a single oxygen-atom vacancy with acetonitrile bound to the axial coordination site, ([V_6_O_6_(OEt)_12_(MeCN)]^0^, **4-MeCN**). This hypothesis was confirmed *via* independent synthesis of **4-MeCN** (Fig. S16,† see Experimental for details). Further support for fluxional coordination of OPR_3_ was obtained by ^1^H NMR analysis of **4-OPMe_2_Ph** in a non-coordinating solvent (DCM-d_2_, Fig. S17†). This spectrum revealed a single set of paramagnetic resonances, with a five-peak pattern similar to that of **4-OPMe_3_** (for full characterization of **4-MeCN** and **4-OPMe_2_Ph** see Fig. S14, S15, S18 and Tables S9, S10†).

While OAT was possible for complex **3** using PMe_2_Ph as a reductant, the steric bulk imparted by the alkoxide ligands to the vanadyl moiety at the surface of the POV-ethoxide cluster significantly affected the reaction rate (4 days) and promoted phosphine oxide dissociation in the presence of coordinating solvents. Attempts to reduce complex **3** with more sterically encumbered tertiary phosphanes (PMePh_2_, PPh_3_) resulted in limited OAT after prolonged heating (Fig. S19 and S20†). A similar lack of reactivity was observed when OAT was attempted from the POV-*propoxide* analogue of the hexavanadate assemblies [V_6_O_7_(O^*n*^Pr)_12_]^0^ (Fig. S21†). These results highlight the sensitivity of this reaction to the steric bulk of both phosphane and POV-alkoxide, indicating that the efficiency of OAT can be influenced by limiting the accessibility of the vanadyl moieties.

### Selective deoxygenation of styrene oxide using [V_6_O_6_(OR)_12_OPMe_3_]^0^ (R = Me, Et)

While the reactivity of POMs in an oxidative capacity is well understood, comparatively less is known about their ability to mediate *reductive* organotransformations.^23^,^63^–^67^ As such, with the reduced POV-alkoxide structures in hand, we shifted our focus toward elucidation of new reductive transformations using these oxygen-atom deficient clusters. In particular, we became interested in the deoxygenation of epoxides, resulting in the formation of an alkene. The described reaction is an important chemical process in organic synthesis, as oxirane rings are commonly used for the chemical protection of an alkene fragment.^68^–^71^ Indeed, deprotection reactions of epoxides are often performed through stoichiometric homogeneous processes that produce large amounts of toxic by-products.^70^,^72^,^73^ Only recently have nonprecious, metal-oxide derived materials (*e.g.* TiO_2_) been implemented for the deoxygenation of this class of substrates.^74^ Notably, mechanistic investigations have revealed that O-atom vacancy formation at the surface of metal-oxide materials plays a key role in the activation of substrate.

**Scheme 4:**
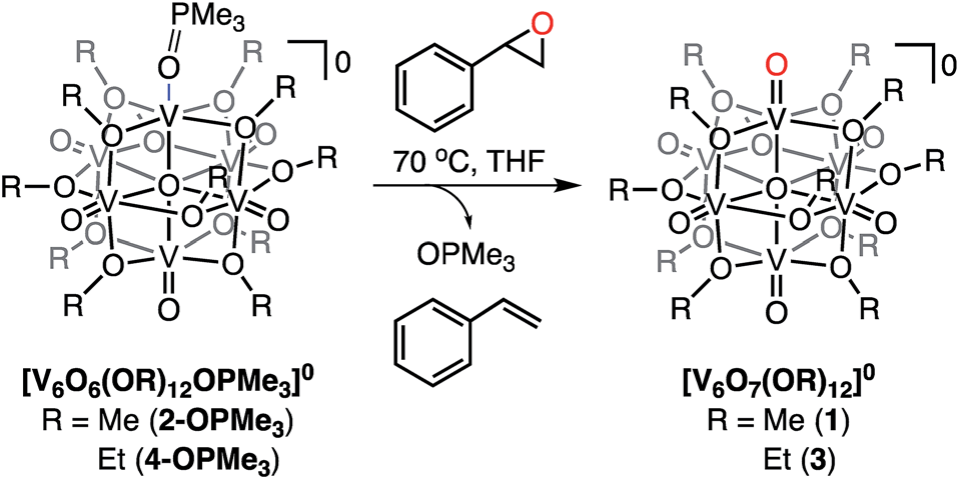
Deoxygenation of styrene oxide using (OPMe_3_)-bound methoxide and ethoxide POV-alkoxide clusters.

Given that our reduced POV-alkoxide clusters bear oxygen-atom deficient sites, we hypothesized that similar C–O bond activation reactions could afford epoxide reduction at the reactive surface sites of the Lindqvist core. Thus, we opted to investigate the reactivity of the (OPMe_3_)-bound methoxide cluster, **2-OPMe_3_**, with an equivalent of styrene oxide (Scheme 4 and Table 2). Indeed, exposure of the substrate to **2-OPMe_3_** at 70 °C resulted in complete consumption of styrene oxide after 2 days (Fig. S22†). However, only 46% of the deoxygenated product, styrene, was formed (^1^H NMR yield *vs.* propylene carbonate as an internal standard, Fig. S22†). Further analysis of the paramagnetic region of the ^1^H NMR spectrum revealed a mixture of resonances that correspond to both the reduced and oxidized vanadate clusters, complexes **2-OPMe_3_** and **1**, respectively (Fig. S23†). The presence of unreacted, reduced cluster, coupled with the complete consumption of styrene oxide, suggests that substrate decomposition (polymerization) is competitive with OAT, preventing full conversion of the reduced POV-alkoxide to **1**.^75^–^77^ To verify this hypothesis, we added a subsequent equivalent of styrene oxide to the crude reaction mixture, affording further oxidation of **2-OPMe_3_**. This experiment confirms that OAT from styrene oxide is hindered by substrate degradation under the prescribed reaction conditions (Fig. S24†).

**Table 2:** Deoxygenation of styrene oxide with **2-OPMe_3_** and **4-OPMe_3_**^*a*^ ^,^^*b*^

Complex	Time (h)	Styrene oxide^*c*^ (%)	Styrene^*c*^ (%)
**2-OPMe_3_**	48	0%	46%
**4-OPMe_3_**	4	22%	83%

^*a*^Deoxygenation was carried out using the (OPMe_3_)-bound clusters (1 equiv.) and styrene oxide (1.1 equiv.).

^*b*^GCMS analysis revealed selective formation of styrene as product (no evidence for the formation of 1-phenethanol or 2-phenethanol).

^*c*^Yield determined by ^1^H NMR spectroscopy *vs.* an internal standard in CD_2_Cl_2_ (propylene carbonate, 1 equiv., Fig. S22–S26).

Intrigued by whether we could extend OAT to the reduced longer-alkoxy bridged cluster, we added styrene oxide (1 equiv.) to **4-OPMe_3_**. In contrast to the results obtained with the methoxide-bridged species, complete consumption of **4-OPMe_3_** was observed by paramagnetic ^1^H NMR spectroscopy after 4 h at 70 °C (Scheme 4, Table 2 and Fig. S25†). Quantification of styrene produced from this reaction was confirmed by ^1^H NMR spectroscopy, revealing formation of 83% styrene (*vs.* propylene carbonate as an internal standard, Fig. S26†).

**Scheme 5:**
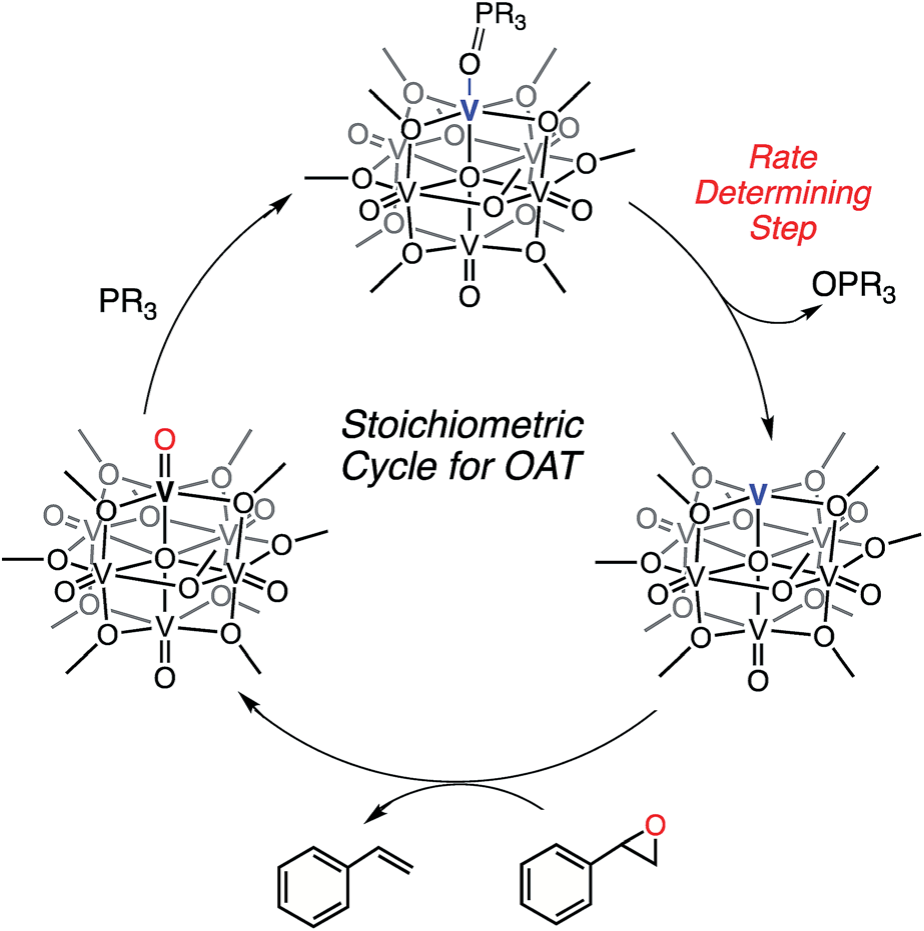
Stoichiometric oxygen atom transfer facilitated by an oxygen-atom vacancy on a POV-alkoxide cluster.

The difference in rate and yield of the reaction of styrene oxide with the two reduced POV-alkoxide clusters (**2-OPMe_3_** and **4-OPMe_3_**) suggests that the steric bulk of the bridging alkoxide moieties around the active V^III^ centre influence OAT. In contrast to the changes in rate observed in the case of oxygen-atom vacancy formation with tertiary phosphanes, the reaction of the POV-ethoxide cluster and styrene oxide is completed in less than 10% of the time required for its methoxide congener (Table 2). We justify these observations through consideration of the lability of the dative phosphine oxide ligands in the case of the methoxide- and ethoxide-bridged POV clusters. For deoxygenation of the styrene oxide to occur, the phosphine oxide ligand must dissociate from the reduced vanadium centre, resulting in the formation of a coordinatively unsaturated metal ion (Scheme 5). We recall that in the case of complex **4-OPMe_2_Ph**, the OPR_3_ ligand readily dissociates from the cluster in the presence of coordinating solvents (substrates). The observed increase in rate of epoxide deoxygenation with **4-OPMe_3_** (as compared to **2-OPMe_3_**) can therefore be attributed to an increased rate of phosphine oxide dissociation from the POV-ethoxide scaffold. Thus, we can conclude that the rate limiting step in alkene formation is the generation of the coordinatively unsaturated vanadium(iii) centre *via* dissociation of OPR_3_.

Prior to this work, only one example of the reductive reactivity of a series of homometallic POMs containing a spectroscopically well-defined defect site had been reported.^36^ The investigation pertaining to the oxidation of these reduced polyoxotungstate clusters, [XW_12_O_37_(OH_2_)_3_]^*n*–^ (X = H_2_, B, Si; *n* = 4–6) detailed the reactivity of these systems with OAT reagents, including Ph_3_AsO, PhNO, and R_2_SO (R = Me, Ph). In all cases, OAT is facilitated by substrates bearing oxygen-heteroatom multiple bonds, resulting in oxidation of the cluster *via* the well-defined multielectron chemistry of the substrate. In contrast, the work presented here summarizes the homogeneous deoxygenation of an epoxide by a reduced homometallic metal oxide cluster, resulting in formation of the corresponding alkene. The reduction of styrene oxide highlights a rare example of the activation of a purely organic substrate by a structurally characterized, oxygen-deficient POM. This work, taken in conjunction with the reductive transformations of homometallic polyoxotungstate clusters summarized in the 1980s by Piepgrass and Pope, prompts further investigation into the ability of reduced POM architectures bearing oxygen-atom defect sites to facilitate reductive transformations of relevance to organic chemistry and small molecule activation.^36^,^37^

## Conclusions

We have demonstrated an alternative route for O-atom vacancy formation at the surface of alkoxy-bridged POV clusters *via* OAT to a series of tertiary phosphanes (PR_3_ = PMe_3_, PMe_2_Ph, PMePh_2_, PPh_3_). While the reactivity of polyoxometalates with phosphane substrates has been reported previously,^29^,^30^,^53^ evidence suggesting an important role of surface-bound water ligands has created ambiguity as to whether oxygen-atom vacancies are truly formed during the course of the reaction.^55^,^78^ In contrast, this work offers clear indication of OAT between cluster and substrate, resulting in the formation of mono-vacant POV-alkoxide clusters with a datively coordinated phosphine oxide ligand bound to the oxygen deficient moiety. The rigorously nonaqueous reaction environment imparted to the polyoxovanadate by its bridging alkoxide ligands eliminates the possibility of proton/water assisted phosphane oxidation. Structural and spectroscopic characterization of the reduced clusters, [V_6_O_6_(OR)_12_OPR′_3_]^0^, provides unambiguous support for complete OAT from the polyoxometalate to the organophosphane substrate.

Furthermore, we have presented an investigation of structure–function relationships, elucidating the role of sterics and electronics on OAT between POV-alkoxide clusters and tertiary phosphanes. While similar investigations regarding the reactivity of phosphanes with mononuclear metal–oxo complexes have been reported previously, this work presents the first example of such a study with polynuclear assemblies. The implication of nucleophilicity and cone angle of the phosphane reductant on OAT was probed, both by varying substituents of the phosphane as well as through the investigation of the reactivity of more sterically encumbered POV-alkoxide architectures. These results reveal a positive correlation between the sterics of both substrate and metal-oxide cluster and reaction time. Collectively, our observations indicate that increased bulk limits the rate of OAT between the metal-oxide cluster and phosphane. In sum, this manuscript provides key insight into the mechanism of formation of O-deficient sites in POV-alkoxide clusters.

Finally, we present an example of a *reductive* organotransformation mediated by a polyoxometalate cluster. Deoxygenation of styrene oxide *via* OAT to the (OPMe_3_)-bound scaffolds, [V_6_O_6_(OR)_12_OPMe_3_]^0^ (R = Me, Et), presents the first example of OAT to a reduced, homometallic polyoxometalate system. Comparative investigations between the reactivity of methoxide- and ethoxide-bridged clusters provides insight into the mechanism of deoxygenation by the cluster. The higher yield of styrene observed in the case of the (OPMe_3_)-bound *ethoxide* species suggests that increased lability of the phosphine oxide ligand lowers the kinetic barrier for epoxide deoxygenation. Thus, we propose that styrene formation proceeds *via* a disassociate mechanism, with the rate limiting step being disassociation of the phosphine oxide ligand from the surface of the cluster (Scheme 5). Future work investigating the reactivity of the reduced POV-alkoxide clusters will expand upon the deoxygenation of various kinds of epoxides including less activated aliphatic substrates.

## Experimental

### General considerations

All manipulations were carried out in the absence of water and dioxygen using standard Schlenk techniques, or in a UniLab MBraun inert atmosphere drybox under a dinitrogen atmosphere except where specified otherwise. All glassware was oven-dried for a minimum of 3 hours and cooled in an evacuated antechamber prior to use in the drybox. Anhydrous tetrahydrofuran was purchased from Sigma-Aldrich and stored over activated 3 Å molecular sieves. All other solvents were dried and deoxygenated on a Glass Contour System (Pure Process Technology, LLC) and stored over activated 3 Å molecular sieves purchased from Fisher Scientific prior to use. [V_6_O_7_(OMe)_12_]^0^ (**1**),^14^ [V_6_O_6_(OMe)_12_]^0^ (**2**),^35^ [V_6_O_7_(OEt)_12_]^0^ (**3**),^15^ and [V_6_O_7_(O^*n*^Pr)_12_]^0^,^62^ were prepared according to published procedures. Trimethlyphosphine, dimethylphenylphosphine, triphenylphosphine, and propylene carbonate were purchased from Sigma Aldrich and used as received. Diphenylmethylphosphine was purchase from Alfa Aesar and used as received.

All ^1^H NMR spectra were recorded at 500 and 400 MHz on Bruker DPX-500 and Bruker DPX-400 MHz spectrometers locked on the signal of deuterated solvents. All chemical shifts were reported relative to the peak of residual H signal in deuterated solvents. CD_3_CN and CD_2_Cl_2_ were purchased from Cambridge Isotope Laboratories, degassed by three freeze–pump–thaw cycles, and stored in the drybox over activated 3 Å molecular sieves. THF-d_8_ was purchased from Cambridge Isotope Laboratories and stored over activated 3 Å molecular sieves. Infrared (FT-IR, ATR) spectra of complexes were recorded on a Shimadzu IRAffinity-1 Fourier transform infrared spectrophotometer and are reported in wavenumbers (cm^–1^). Electronic absorption measurements were recorded at room temperature in anhydrous acetonitrile in a sealed 1 cm quartz cuvette with an Agilent Cary 60 UV-vis spectrophotometer. Mass spectrometry analyses were performed on an Advion Expression^L^ Compact Mass Spectrometer equipped with an electrospray probe and an ion-trap mass analyser. Direct injection analysis was employed in all cases with a sample solution in acetonitrile. Cyclic voltammetry experiments were recorded with a Bio-Logic SP200 potentiostat/galvanostat and the EC-Lab software suite. All measurements were performed in a three electrode system cell configuration that consisted of a glassy-carbon (*ø* = 3.0 mm) as working electrode (CH Instruments, USA), a Pt wire as the counter electrode (CH Instruments, USA), and an Ag/Ag^+^ non-aqueous reference electrode with 0.01 M AgNO_3_ in 0.05 M [^*n*^Bu_4_N][PF_6_] in acetonitrile (BASi, USA). All electrochemical measurements were performed at room temperature in a N_2_-filled drybox. Anhydrous dichloromethane that contained [^*n*^Bu_4_N][PF_6_] was used as the electrolyte solution. Gas chromatography-mass spectrometry analyses were performed on a Shimadzu GCMS QP 2010.

Single crystals of **2-OPMe_3_**, **2-OPMe_2_Ph**, **2-OPMePh_2_**, **2-OPPh_3_** and **4-OPMe_3_** were mounted on the tip of a thin glass optical fiber (goniometer head) and mounted on a XtaLab Synergy-S Dualflex diffractometer equipped with a HyPix-6000HE HPC area detector for data collection at 100.00(10)–192.99(10) K, respectively. The structure was solved using SHELXT-2018/2 (ref. 79) and refined using SHELXL-2018/3.^60^ Elemental analyses were performed on a PerkinElmer 2400 Series II Analyzer, at the CENTC Elemental Analysis Facility, University of Rochester.

### Synthesis of [V_6_O_6_(OCH_3_)_12_OP(CH_3_)_3_] (**2-OPMe_3_**)

In a glovebox, a 15 mL pressure vessel was charged with [V_6_O_7_(OCH_3_)_12_] (**1**) (0.051 g, 0.065 mmol) and 10 mL tetrahydrofuran. A 1.0 M solution of trimethylphosphine in tetrahydrofuran (0.26 mL, 0.26 mmol, 4 equiv.) was added dropwise to the reaction mixture and the pressure vessel was sealed, removed from the glovebox, and placed in an oil bath and set to stir for 19 hours at 70 °C. After this time, the reaction vessel was brought back into the glovebox, and solvents were removed under reduced pressure to give a green/brown solid. The remaining residual solid was stirred in pentane (10 mL) overnight and then filtered. The solid was washed with excess pentane (5 mL, 3×) until the filtrate ran clear. The solid was then extracted with tetrahydrofuran (2 mL, 3×), and volatiles were removed under vacuum to yield the product, **2-OPMe_3_**, as a dark green solid (0.047 g, 0.055 mmol, 84%). Crystals suitable for X-ray analysis were grown from slow diffusion of pentane into a concentrated solution of the product in tetrahydrofuran. ^1^H NMR (500 MHz, CD_3_CN) *δ* = 28.38 (fwhh = 690), 23.13 (fwhh = 170 Hz), 19.74 (fwhh = 145), –12.25 (fwhh = 269) ppm. FT-IR (ATR, cm^–1^): 1163 (P=O), 1032 (O–CH_3_), 962 (V=O), 945 (P=O). UV-vis (CH_3_CN): 390 nm (*ε* = 3766 M^–1^ cm^–1^), 1000 nm (*ε* = 655 M^–1^ cm^–1^). Elemental analysis for 
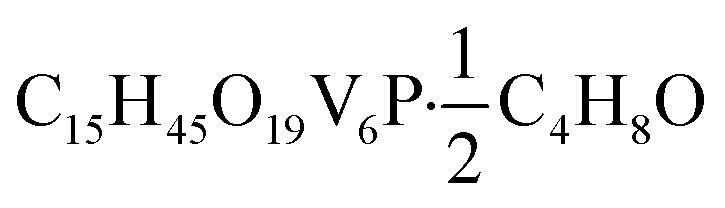
 (MW 902.17 g mol^–1^) calcd (%): C, 22.63; H, 5.47. Found (%): C, 22.50; H, 5.19.

### Synthesis of [V_6_O_6_(OCH_3_)_12_OP(CH_3_)_2_C_6_H_5_] (**2-OPMe_2_Ph**)

In a glovebox, a 15 mL pressure vessel was charged with [V_6_O_7_(OCH_3_)_12_] (**1**) (0.041 g, 0.052 mmol) and 6 mL tetrahydrofuran. Dimethylphenylphosphine (15 μL, 0.10 mmol, 2 equiv.) was added dropwise to the solution. The reaction vessel was sealed, removed from the glovebox, and placed in an oil bath where it was left to stir for 7 hours at 70 °C. After this time, the reaction vessel was brought back into the glovebox, and solvents were removed under reduced pressure to give a brown solid. The remaining residual solid was stirred in pentane (10 mL) for 30 min and then filtered. The solid was washed with excess pentane (5 mL, 3×) until the filtrate ran clear. The solid was then extracted with dichloromethane (2 mL, 3×), and any volatiles were removed under vacuum to yield the product, **2-OPMe_2_Ph**, as a dark brown solid (0.042 g, 0.045 mmol, 87%). Crystals suitable for X-ray analysis were grown from slow diffusion of pentane into a concentrated solution of the product in tetrahydrofuran. ^1^H NMR (400 MHz, CD_3_CN) *δ* = 24.58 (fwhh = 136 Hz), 20.06 (fwhh = 156 Hz), 8.99, 8.54 (fwhh = 68 Hz), 7.69 (fwhh = 68 Hz), 3.42 (fwhh = 136 Hz), –12.52 (fwhh = 372 Hz) ppm. FT-IR (ATR, cm^–1^): 1159 (P=O), 1036 (O–CH_3_), 962 (V=O), 943 (P=O). UV-vis (CH_3_CN): 390 nm (*ε* = 4224 M^–1^ cm^–1^), 1000 nm (*ε* = 721 M^–1^ cm^–1^). Elemental analysis for C_20_H_47_O_19_V_6_P (MW 928.20 g mol^–1^) calcd (%): C, 25.88; H, 5.10. Found (%): C, 26.08; H, 4.92.

### Synthesis of [V_6_O_6_(OCH_3_)_12_OPCH_3_(C_6_H_5_)_2_] (**2-OPMePh_2_**)

In a glovebox, a 15 mL pressure vessel was charged with [V_6_O_7_(OCH_3_)_12_] (**1**) (0.042 g, 0.053 mmol) and 6 mL tetrahydrofuran. Diphenylmethylphosphine (0.04 mL, 0.22 mmol, 4 equiv.) was added dropwise to the solution. The reaction vessel was sealed, removed from the glovebox, and placed in an oil bath where it was left to stir for 28 hours at 70 °C. After this time, the reaction vessel was brought back into the glovebox, and solvents were removed under reduced pressure to give a brown-green oil. The remaining oil was stirred in pentane (10 mL) for 30 min and then filtered. The solid was washed with excess pentane (5 mL, 3×) until the filtrate ran clear. The solid was then extracted with dichloromethane (2 mL, 3×), and any volatiles were removed under reduced pressure to yield the product, **2-OPMePh_2_**, as a dark brown solid (0.044 g, 0.043 mmol, 84%). Crystals suitable for X-ray analysis were grown from slow diffusion of pentane into a concentrated solution of the product in tetrahydrofuran. ^1^H NMR (400 MHz, CD_3_CN) *δ* = 25.13 (fwhh = 172 Hz), 20.17 (fwhh = 136 Hz), 8.52 (fwhh = 36 Hz), 7.71, 7.57, 3.42, –12.52 (fwhh = 304 Hz) ppm. FT-IR (ATR, cm^–1^): 1159 (P=O), 1036 (O–CH_3_), 1013 (P=O), 964 (V=O). UV-vis (CH_3_CN): 390 nm (*ε* = 3195 M^–1^ cm^–1^), 1000 nm (*ε* = 546 M^–1^ cm^–1^). Elemental analysis for C_25_H_49_O_19_V_6_P (MW 990.27 g mol^–1^) calcd (%): C, 30.32; H, 4.99. Found (%): C, 30.45; H, 4.67.

### Synthesis of [V_6_O_6_(OCH_3_)_12_OP(C_6_H_5_)_3_] (**2-OPPh_3_**)

In a glovebox, a 15 mL pressure vessel was charged with [V_6_O_7_(OCH_3_)_12_] (**1**) (0.048 g, 0.055 mmol), triphenylphosphine (0.061 g, 0.233 mmol, 4 equiv.), and 10 mL tetrahydrofuran. The reaction vessel was sealed, removed from the glovebox, and placed in an oil bath where it was left to stir for 15 days at 70 °C. After this time, the reaction vessel was brought back into the glovebox, and solvents were removed under reduced pressure to give a brown solid. The solid was stirred in pentane (10 mL) overnight and then filtered. The remaining solid was washed with excess pentane (5 mL, 3×) until the filtrate ran clear. The solid was then extracted with dichloromethane (2 mL, 3×), and any volatiles were removed under vacuum to yield the product, **2-OPPh_3_**, as a dark brown solid (0.055 g, 0.052 mmol, 82%). Crystals suitable for X-ray analysis were grown from slow diffusion of pentane into a concentrated solution of the product in tetrahydrofuran. ^1^H NMR (400 MHz, CD_3_CN) *δ* = 25.62 (fwhh = 360 Hz), 20.39 (fwhh = 280 Hz), 8.49, 7.37, –13.06 (fwhh = 520 Hz) ppm. FT-IR (ATR, cm^–1^): 1157 (P=O), 1040 (O–CH_3_), 966 (V=O). UV-vis (CH_3_CN): 390 nm (*ε* = 2298 M^–1^ cm^–1^), 1000 nm (*ε* = 467 M^–1^ cm^–1^). Elemental analysis for 
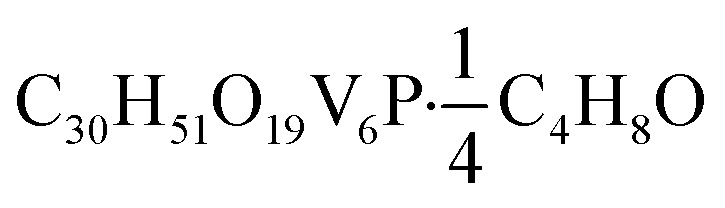
 (MW 1070.35 g mol^–1^) calcd (%): C, 34.79; H, 4.99. Found (%): C, 35.09; H, 5.01.

### Synthesis of [V_6_O_6_(OC_2_H_5_)_12_OP(CH_3_)_3_] (**4-OPMe_3_**)

In a glovebox, a 15 mL pressure vessel was charged with [V_6_O_7_(OC_2_H_5_)_12_]^0^ (**3**) (0.122 g, 0.128 mmol), trimethylphosphine (1 M in tetrahydrofuran, 0.51 mL, 0.51 mmol, 4 equiv.), and 12 mL tetrahydrofuran. The reaction vessel was sealed, removed from the glovebox, and placed in an oil bath where it was left to stir for 4 days at 70 °C. After this time, the reaction vessel was brought back into the glovebox, and volatiles were removed under reduced pressure to give a green-brown solid. The solid was extracted with acetonitrile (5 mL, 3×), and any volatiles were removed under vacuum. The solid was extracted with diethyl ether (5 mL, 3×), and any volatiles were removed under vacuum to yield the product, **4-OPMe_3_**, as a green-brown solid (0.068 g, 0.066 mmol, 52%). The product can be further purified *via* recrystallization from slow diffusion of pentane into a concentrated solution of **4-OPMe_3_** in tetrahydrofuran (0.040, 0.038 mmol, 30%). ^1^H NMR (400 MHz, CD_3_CN): *δ* = 29.16 (fwhh = 272 Hz), 22.08 (fwhh = 200 Hz), 4.69 (fwhh = 120 Hz), 2.26 (fwhh = 64 Hz), –0.53 (fwhh = 76 Hz), –2.41 (fwhh = 84 Hz), –22.62 (fwhh = 424 Hz) ppm. ^1^H NMR (400 MHz, CDCl_3_): *δ* = 33.62 (fwhh = 288 Hz), 18.15 (fwhh = 276 Hz), 4.40 (fwhh = 192 Hz), 2.40 (fwhh = 128 Hz), –0.02 (fwhh = 124 Hz), –3.03 (fwhh = 144 Hz), –22.43 (fwhh = 536 Hz) ppm. FT-IR (ATR, cm^–1^): 1171 (P=O), 1043 (O–C_2_H_5_), 964 (V=O), 943 (P=O). UV-vis (CH_3_CN): 396 nm (*ε* = 4300 M^–1^ cm^–1^), 1000 nm (*ε* = 610 M^–1^ cm^–1^). UV-vis (CH_2_Cl_2_): 394 nm (*ε* = 4010 M^–1^ cm^–1^), 1000 nm (*ε* = 628 M^–1^ cm^–1^). Elemental analysis for C_27_H_69_O_19_V_6_P (MW 1034.45 g mol^–1^) calcd (%): C, 31.35; H, 6.72. Found (%): C, 31.75; H, 6.68.

### Synthesis of [V_6_O_6_(OC_2_H_5_)_12_OP(CH_3_)_2_C_6_H_5_] (**4-OPMe_2_Ph**)

In a glovebox, two 15 mL pressure vessels were charged with [V_6_O_7_(OC_2_H_5_)_12_] (**3**) (0.102 g, 0.106 mmol), dimethylphenylphosphine (0.062 g, 0.44 mmol, 4 equiv.), and 12 mL tetrahydrofuran. The reaction vessels were sealed, removed from the glovebox, and placed in an oil bath where they were left to stir for 4 days at 70 °C. After this time, the reaction vessels were brought back into the glovebox, the solutions were united, and volatiles were removed under reduced pressure to give a green-brown solid. The solid was extracted with acetonitrile (5 mL, 3×), and any volatiles were removed under vacuum. The solid was extracted with diethyl ether (5 mL, 3×), and any volatiles were removed under vacuum to yield the product, **4-OPMe_2_Ph**, as a dark brown solid (0.061 g, 0.055 mmol, 26%). The product can be further purified *via* recrystallization from slow diffusion of pentane into a concentrated solution of **4-OPMe_2_Ph** in tetrahydrofuran (0.027, 0.025 mmol, 12%). These crystals were suitable for X-ray analysis. ^1^H NMR (400 MHz, CD_2_Cl_2_) *δ* = 32.18 (fwhh = 288 Hz), 19.09 (fwhh = 356 Hz), 8.54, 8.33 (fwhh = 72 Hz), 7.54 (fwhh = 52 Hz), 3.82 (fwhh = 172 Hz), 2.55 (fwhh = 128 Hz), –0.14 (fwhh = 116 Hz), –2.86 (fwhh = 124 Hz), –22.05 (fwhh = 556 Hz) ppm. FT-IR (ATR, cm^–1^): 1157 (P=O), 1040 (O–CH_3_), 964 (V=O), 934 (P=O). UV-vis (CH_2_Cl_2_): 394 nm (*ε* = 4710 M^–1^ cm^–1^), 1000 nm (*ε* = 638 M^–1^ cm^–1^). Elemental analysis for C_32_H_71_O_19_V_6_P (MW 1096.51 g mol^–1^) calcd (%): C, 35.05; H, 6.53. Found (%): C, 35.43; H, 6.39.

### Synthesis of [V_6_O_6_(OC_2_H_5_)_12_(CH_3_CN)] (**4-MeCN**)

The synthesis of **4-MeCN** was modelled after that previously reported for complex **2-MeCN**.^35^ In a glovebox, two 20 mL scintillation vials were charged with [V_6_O_7_(OC_2_H_5_)_12_] (**3**) (0.101 g, 0.106 mmol) and 4 mL tetrahydrofuran. In separate vials, VMes_3_(THF) (0.0559 g, 0.117 mmol, 1.1 equiv.) was dissolved in 8 mL tetrahydrofuran, and these solutions were added dropwise to the corresponding **3** solution. The reaction mixtures were heated to 50 °C for an hour, affording a red-brown solution. The reaction mixtures were united, and the volatiles were removed under vacuum. The resulting brown solid was washed with acetonitrile (4 mL), then washed with pentane until the pentane went from red-orange to yellow (12 mL, 6×). The solid was extracted with acetonitrile (12 mL × 6) and filtered over a bed of Celite (1 cm) on a medium-porosity glass frit. The volatiles were removed under vacuum to yield the product, **4-MeCN**, as a dark brown solid (0.099 g, 0.100 mmol, 47%). ^1^H NMR (400 MHz, CD_3_CN): *δ* = 30.53 (fwhh = 476 Hz), 20.74 (fwhh = 304 Hz), 3.07, –0.21 (fwhh = 148 Hz), –2.55 (fwhh = 160 Hz), –24.21 (fwhh = 548 Hz) ppm. FT-IR (ATR, cm^–1^): 1040 (O–C_2_H_5_), 964 (V=O). UV-vis (CH_3_CN): 394 nm (*ε* = 2740 M^–1^ cm^–1^), 1000 nm (*ε* = 407 M^–1^ cm^–1^). UV-vis (CH_2_Cl_2_): 394 nm (*ε* = 2790 M^–1^ cm^–1^), 1000 nm (*ε* = 417 M^–1^ cm^–1^). Elemental analysis for 
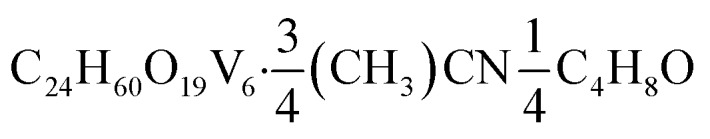
 (MW 991.20 g mol^–1^) calcd (%): C, 32.11; H, 6.53; N, 1.06. Found (%): C, 32.33; H, 6.55; N, 1.18.

### Oxygen atom transfer from styrene oxide to **2-OPMe_3_**

In a glovebox, a J-young tube was charged with **2-OPMe_3_** (0.016 g, 0.019 mmol, 1 equiv.), styrene oxide (2.25 μL, 0.020 mmol, 1.1 equiv.), propylene carbonate (1.50 μL, 0.018 mmol, 1 equiv.), and 0.7 mL CD_2_Cl_2_. The J-young reaction was placed in a 70 °C oil bath and removed for time points. ^1^H NMR yields were determined by integration against an internal standard, propylene carbonate.

### Oxygen atom transfer from styrene oxide to **4-OPMe_3_**

In a glovebox, a J-young tube was charged with **4-OPMe_3_** (0.204 g, 0.020 mmol, 1 equiv.), styrene oxide (2.50 μL, 0.022 mmol, 1.1 equiv.), propylene carbonate (1.75 μL, 0.021 mmol, 1 equiv.), and 0.7 mL CD_2_Cl_2_. The J-young reaction was placed in a 70 °C oil bath and removed for time points. ^1^H NMR yields were determined by integration against an internal standard, propylene carbonate.

## Conflicts of interest

There are no conflicts to declare.

## Supplementary Material

Supplementary information

Crystal structure data
