# Supplementary material for: Oxygen atom transfer with organofunctionalized polyoxovanadium clusters: O-atom vacancy formation with tertiary phosphanes and deoxygenation of styrene oxide
Source: Chem Sci. 2019 Jul 15;10(34):8035–45. doi: 10.1039/c9sc02882j (PMC6837047; doi:10.1039/c9sc02882j)
Supplement: Supplementary file 1 [file SC-010-C9SC02882J-s001.pdf]

Electronic Supplementary Material (ESI) for Chemical Science.
This journal is © The Royal Society of Chemistry 2019

Oxygen atom transfer with organofunctionalized polyoxovanadium clusters: O-atom vacancy formation with tertiary phosphanes and deoxygenation of styrene oxide

Brittney E. Petel[a], Rachel L. Meyer[a], William W. Brennessel[a], Ellen M. Matson*[a]

[a] Department of Chemistry, University of Rochester, Rochester, NY 14627, USA.

Corresponding author email: matson@chem.rochester.edu

Supporting Information Table of Contents:

Figure S1. ^{1}H NMR spectra of optimization for **2-OPMe$_3$**……S3
Figure S2. ^{1}H NMR spectra of **1**, **2-MeCN**, and **2-OPMe$_3$**……S3
Figure S3. ESI-MS (+)ve of **2-OPMe$_3$**……S4
Figure S4. Infrared spectra of **2-OPMe$_3$**……S4
Figure S5. Electronic absorption spectra of **2-OPMe$_3$**……S5
Table S1. Crystallographic parameters of **2-OPMe$_3$**……S6
Table S2. Bond valence sum calculations for **2-OPMe$_3$**……S6
Figure S6. Molecular structure of **2-OPMe$_3$**……S7
Table S3. Selected bond lengths of **2-OPMe$_3$**……S7
Figure S7. ^{1}H NMR Spectrum of **2-OPMe$_3$**, **2-OPMe$_2$Ph**, **2-OPMePh$_2$**, and **2-OPPh$_3$**……S8
Figure S8. ESI-MS (+)ve of **2-OPMe$_2$Ph**……S8
Figure S9. ESI-MS (+)ve of **2-OPMePh$_2$**……S9
Figure S10. ESI-MS (+)ve of **2-OPPh$_3$**……S9
Table S4. Crystallographic parameters of **2-OPMe$_2$Ph**, **2-OPMePh$_2$**, and **2-OPPh$_3$**……S10
Table S5. Selected bond lengths and angles of **2-OPMe$_2$Ph**, **2-OPMePh$_2$**, and **2-OPPh$_3$**……S10
Table S6. Bond valence sum calculations for **2-OPMe$_2$Ph**, **2-OPMePh$_2$**, and **2-OPPh$_3$**……S11
Table S7. Bond vibrations of **2-OPMe$_3$**, **2-OPMe$_2$Ph**, **2-OPMePh$_2$**, and **2-OPPh$_3$**……S11
Table S8. Relationship between nucleophilicity, cone angle, and reaction time……S12
Figure S11. ^{1}H NMR Spectrum of $V_6O_5(OMe)_{12}(OPMe_2Ph)_2$……S12
Figure S12. ESI-MS (+)ve of $V_6O_5(OMe)_{12}(OPMe_2Ph)_2$……S13
Figure S13. ^{1}H NMR Spectrum of **4-OPMe$_3$**……S13
Figure S14. Infrared spectra of **3**, **4-MeCN**, **4-OPMe$_3$**, and **4-OPMe$_2$Ph**……S14
Table S9. Bond vibrations of **4-MeCN**, **4-OPMe$_3$**, and **4-OPMe$_2$Ph**……S14
Figure S15. Electronic absorption spectra of **3**, **4-MeCN**, **4-OPMe$_3$**, and **4-OPMe$_2$Ph**……S15
Figure S16. ^{1}H NMR spectra of **4-MeCN** and **4-OPMe$_2$Ph** in CD_3CN……S15
Figure S17. ^{1}H NMR spectrum of **4-OPMe$_2$Ph** in CD_2Cl_2……S16
Figure S18. Molecular structure of **4-OPMe$_2$Ph**……S16
Table S10. Crystallographic parameters of **4-OPMe$_2$Ph**……S17
Figure S19. ^{1}H NMR spectra of attempted synthesis of **4-OPMePh$_2$**……S18
Figure S20. ^{1}H NMR spectra of attempted synthesis of **4-OPPh$_3$**……S18
Figure S21. ^{1}H NMR spectra of $[V_6O_7(OPr)_{12}]^0$ + PR_3……S19
Figure S22. Diamagnetic ^{1}H NMR spectra of **2-OPMe$_3$** + styrene oxide……S20
Figure S23. Paramagnetic ^{1}H NMR spectra of **2-OPMe$_3$** + styrene oxide……S20
Figure S24. Paramagnetic ^{1}H NMR spectra of **2-OPMe$_3$** + *excess* styrene oxide……S21
Figure S25. Paramagnetic ^{1}H NMR spectra of **4-OPMe$_3$** + styrene oxide……S22
Figure S26. Diamagnetic ^{1}H NMR spectra of **4-OPMe$_3$** + styrene oxide……S22
References……S23

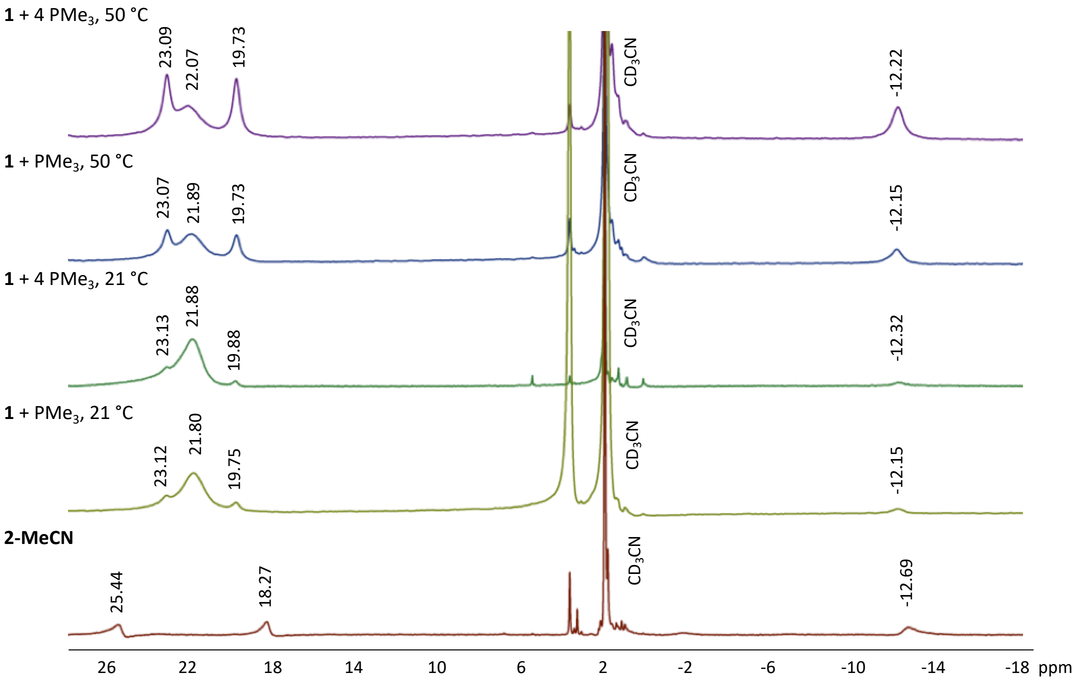

Figure S1. ^{1}H NMR spectra of the reaction mixtures of $[V_6O_7(OMe)_{12}]^0$ (**1**) and PMe_3 (1 or 4 equiv) after stirring at 21 or 50 ºC for 24 hours in THF (see labels for reaction details). All spectra were collected in CD_3CN at 21 ºC.

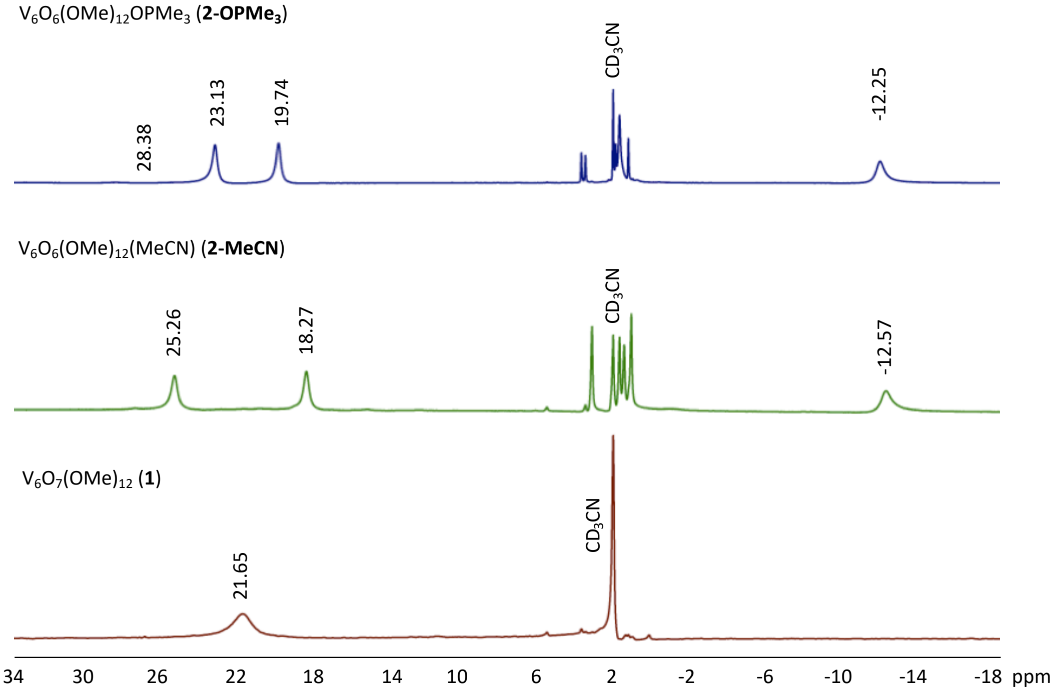

Figure S2. ^{1}H NMR spectrum of **1** (bottom, red), **2-MeCN** (middle, green), and **2-OPMe$_3$** (top, blue) in CD_3CN at 21 ºC.

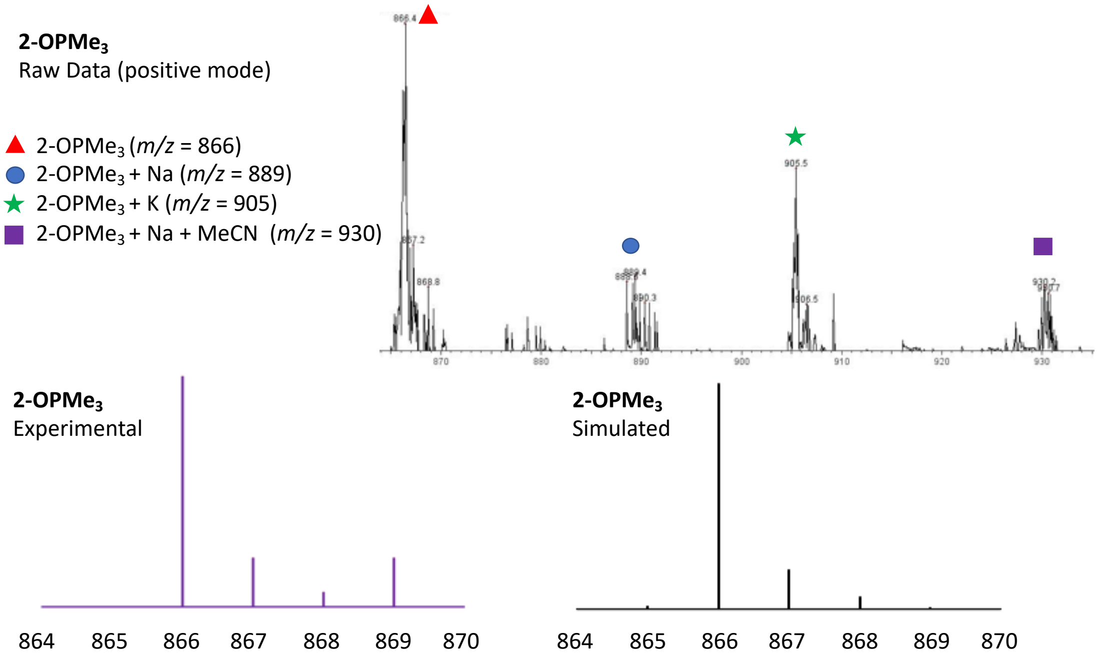

Figure S3. ESI-MS (+)ve of **2-OPMe$_3$** (m/z = 866).

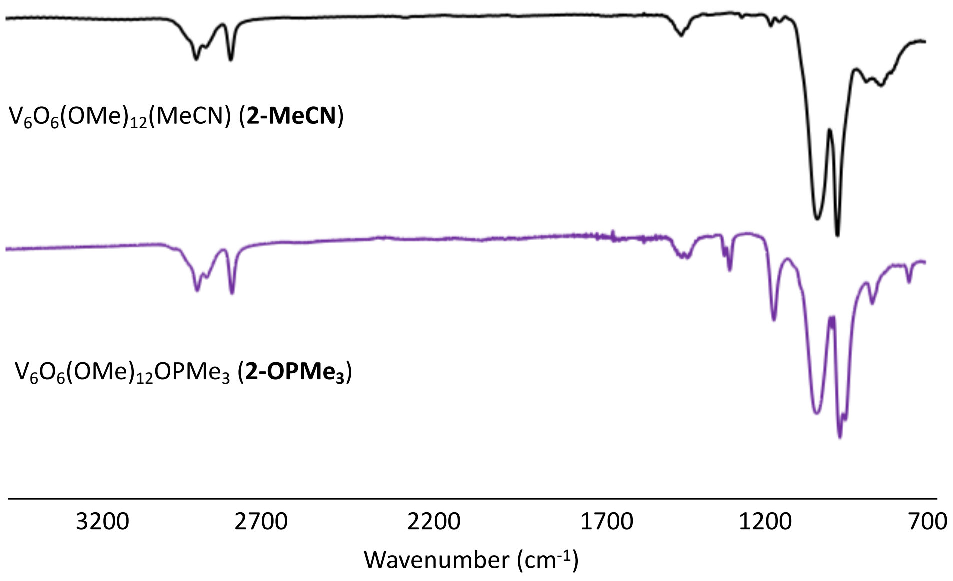

Figure S4. Infrared absorption spectra of **2-MeCN** (top, black), and **2-OPMe$_3$** (bottom, purple). See Table S7 for bond vibrations.

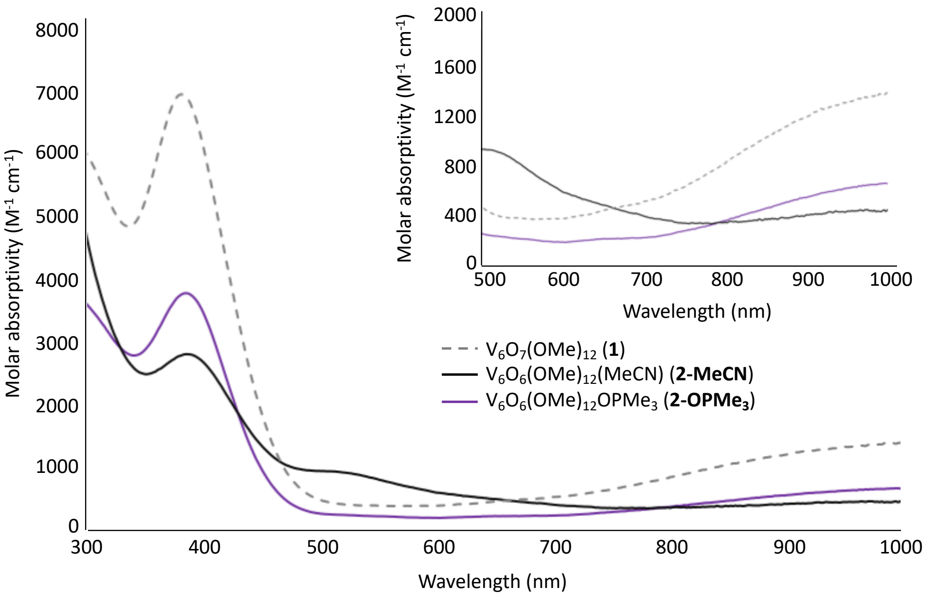

Figure S5. Electronic absorption spectra of complexes **1** (grey dashed), **2-MeCN** (black), and **2-OPMe$_3$** (purple) collected in acetonitrile at 21 ºC.

Table S1. Crystallographic Parameters of **2-OPMe$_3$** (CCDC: 191251)

Empirical formula	$C_{15}H_{45}PO_{19}V_6$	
Formula weight	866.12	
Temperature	192.99(10) K	
Wavelength	1.54184 Å	
Crystal system	Monoclinic	
Space group	P 2_1/c	
Unit cell dimensions	a = 20.7527(3) Å<br>b = 20.6175(3) Å<br>c = 17.6285(3) Å	α = 90°<br>β = 106.417(2)°<br>γ = 90°
Volume	7235.2(2) Å^3	
Z	8	
Reflections collected	62308	
Independent reflections	15129	
Goodness-of-fit on F2	1.048	
Final R indices [I>2sigma(I)]	R1 = 0.0967, wR2 = 0.2638	

Table S2. Bond valence sum calculations for the crystallographically independent vanadium ions in **2-OPMe$_3$** based on X-ray crystallographic data collected at 193 K. Table reflects the results of BVS calculations using V-O bond valence parameters (r_0) for different oxidation states of vanadium.

2-OPMe$_3$	V1	V2	V3	V4	V5	V6
V(III)	**3.102**	4.005	3.970	4.033	4.076	4.471
V(IV)	3.176	**4.100**	**4.065**	**4.129**	**4.173**	4.577
V(V)	3.414	4.370	4.333	4.401	4.446	**4.872**

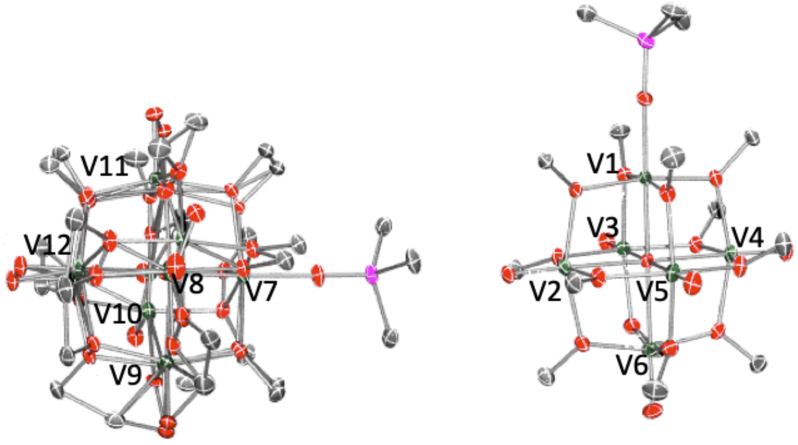

Figure S6. Molecular structures of both independent molecules in the asymmetric unit of **2-OPMe$_3$** shown with 10% probability ellipsoids. Hydrogen atoms removed for clarity. Highly disordered structure (left, atoms V7-V12) is disordered over two positions due to adjacency with its symmetry equivalents. Bond metrics of **2-OPMe$_3$** obtained from the molecule without disorder (right, atoms V1-V6).

Table S3. Selected bond lengths and angles of **2-OPMe$_3$** and comparison to previously reported mono- and di-vacant complexes.

**Bond Distances and Angles**	$V_6O_7(OMe)_{12}$ **(1)**	$V_6O_6(OMe)_{12}OPMe_3$ **(2-OPMe$_3$)**	$V_6O_6(OMe)_{12}OTf$	$V_6O_5(OMe)_{12}(MeCN)_2$
Reference	Hartl, 2005[1]	This work	Matson, 2018[2]	Matson, 2019[3]
V1-O1	--	2.026(5) Å	2.052(8) Å	--
O=P	--	1.467(6) Å	--	--
V1-O$_c$	2.25 Å	2.120(5) Å	2.079(4) Å	2.0666(17) Å<br>2.0760(17) Å
V=O$_t$ (avg.)	1.60 Å	1.592 Å	1.585 Å	1.605 Å
V1-O$_b$-V$_n$ (avg.)	--	~105°	~105°	~105°

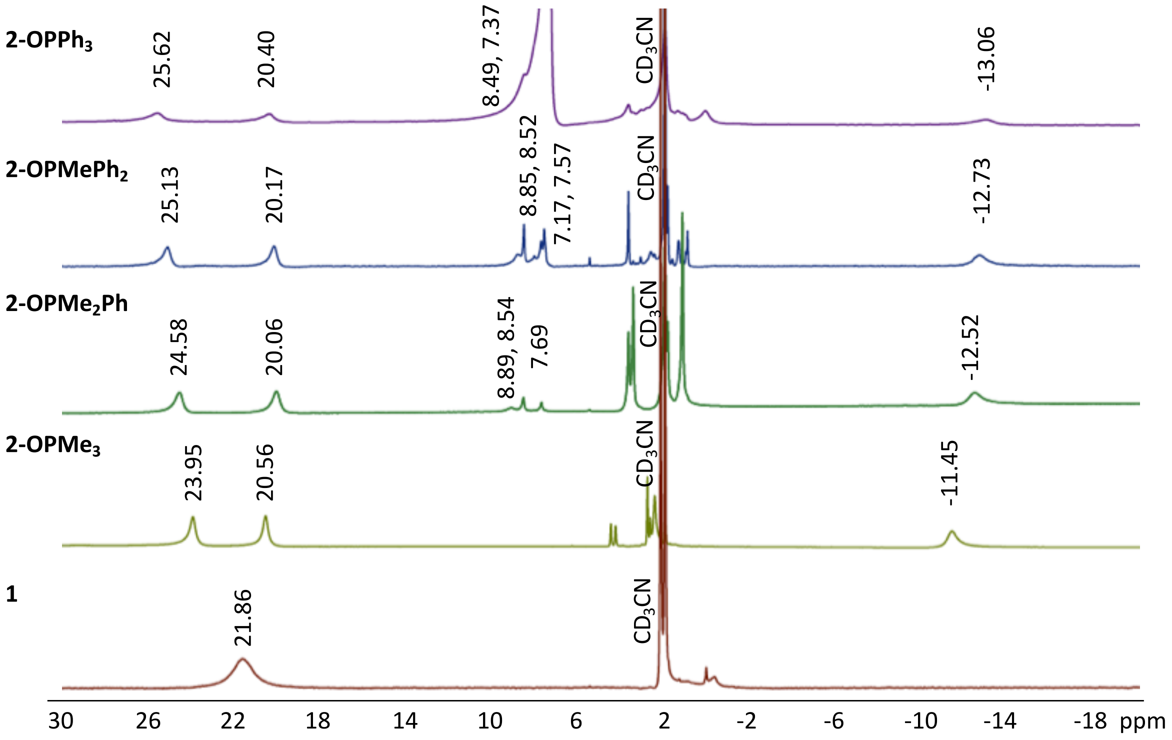

Figure S7. ^{1}H NMR spectrum of **1** (red), **2-OPMe$_3$** (yellow), **2-OPMe$_2$Ph** (green), **2-OPMePh$_2$** (blue) and **2-OPPh$_3$** (purple) in CD_3CN at 21 ºC.

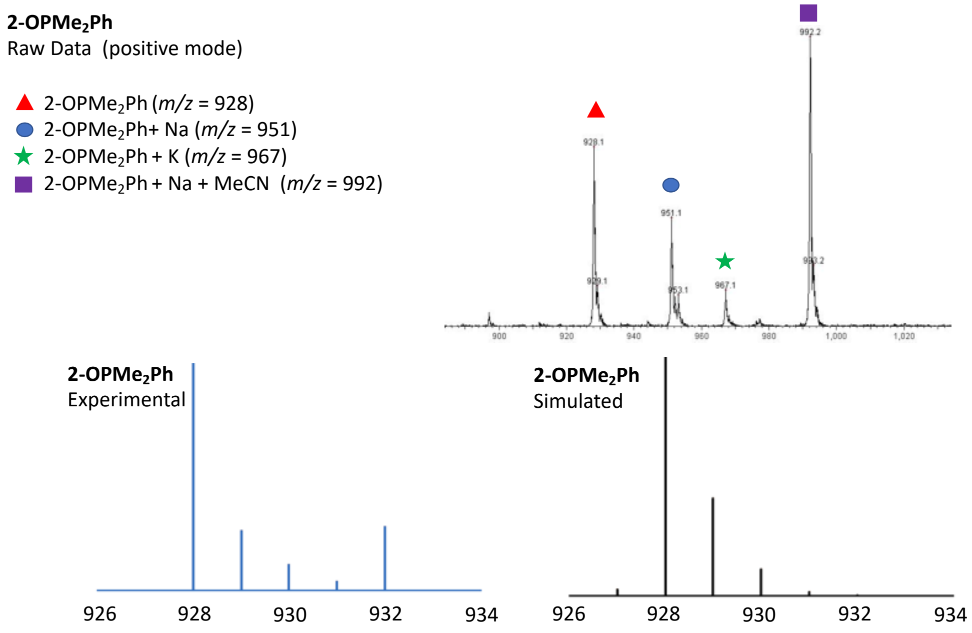

Figure S8. ESI-MS (+)ve of **2-OPMe$_2$Ph** (m/z = 928).

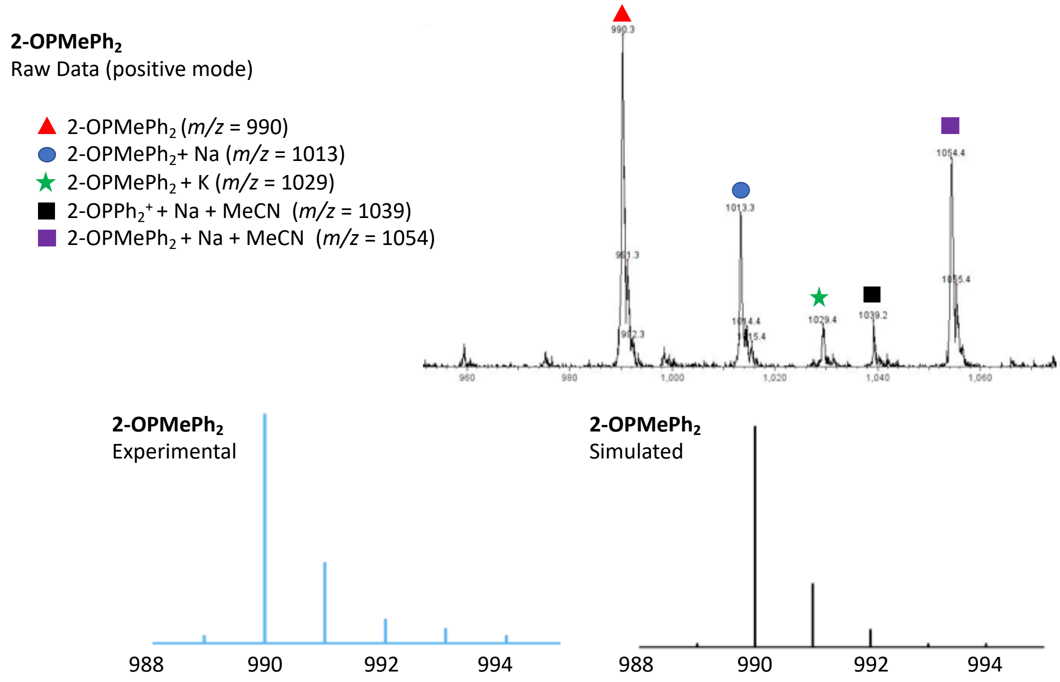

Figure S9. ESI-MS (+)ve of **2-OPMePh$_2$** (m/z = 990).

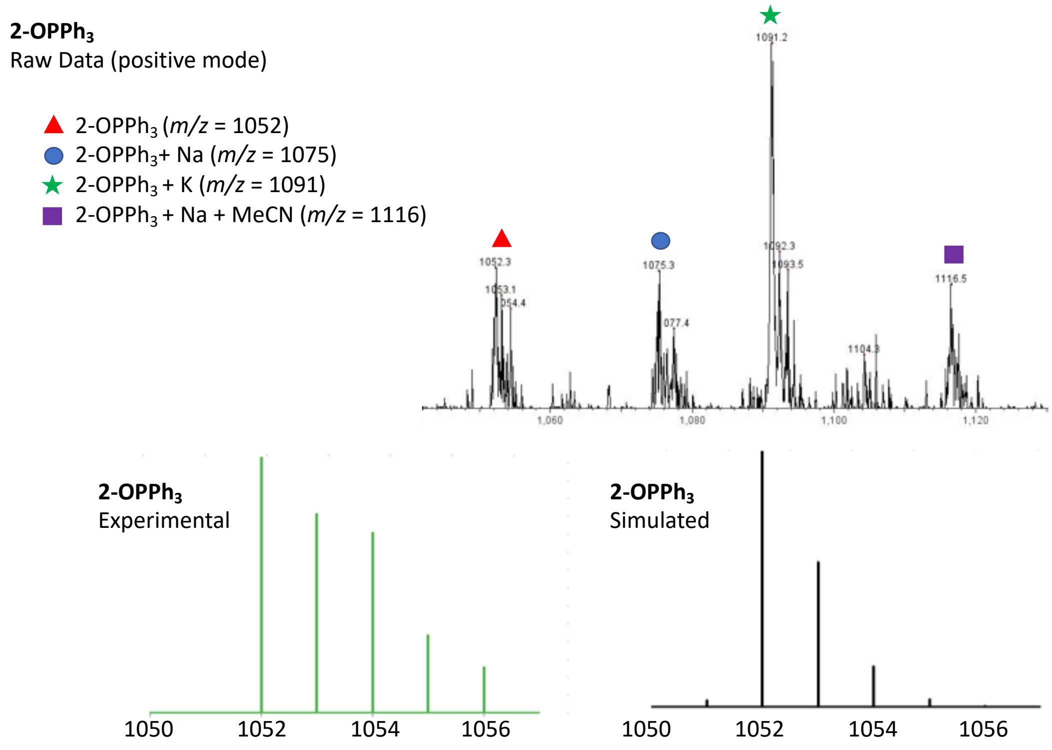

Figure S10. ESI-MS (+)ve of **2-OPPh$_3$** (m/z = 1052).

Table S4. Crystallographic Parameters of **2-OPMe$_2$Ph**, **2-OPMePh$_2$**, and **2-OPPh$_3$** (CCDC: 1914252 – 1914254).

Molecule	**2-OPMe$_2$Ph**	**2-OPMePh$_2$**	**2-OPPh$_3$**
Empirical formula	$C_{20}H_{47}PO_{19}V_6$	$C_{25}H_{49}PO_{19}V_6$	$C_{30}H_{51}PO_{19}V_6$
Formula weight	928.18	990.25	1052.31
Temperature	100.00(10) K	100.0(4) K	100.0(5) K
Wavelength	1.54184 Å	1.54184 Å	1.54184 Å
Crystal system	Monoclinic	Monoclinic	Orthorhombic
Space group	C 2/*c*	$P2_1$	$P2_12_12_1$
Unit cell dimensions	a = 33.3410(3) Å<br>b = 10.76690(10) Å<br>c = 20.2152(2) Å<br>$\alpha = 90^o$<br>$\beta = 92.6080(10)^o$<br>$\gamma = 90^o$	a = 11.0627(8) Å<br>b = 10.8304(7) Å<br>c = 16.9545(12) Å<br>$\alpha = 90^o$<br>$\beta = 108.004(8)^o$<br>$\gamma = 90^o$	a = 13.72880(10) Å<br>b = 16.00950(10) Å<br>c = 18.8692(2) Å<br>$\alpha = 90^o$<br>$\beta = 90^o$<br>$\gamma = 90^o$
Volume	7249.43(12) Å^3	1.931.9(2) Å^3	4147.28(6) Å^3
Z	8	2	4
Reflections collected	34073	27440	38388
Independent reflections	7553	7696	8699
Goodness-of-fit on F2	1.079	1.093	1.090
Final R indices [I>2sigma(I)]	R1 = 0.0414<br>wR2 = 0.1165	R1 = 0.1213<br>wR2 = 0.2832	R1 = 0.0395<br>wR2 = 0.1074

Table S5. Selected bond lengths and angles of **2-OPMe$_2$Ph**, **2-OPMePh$_2$**, and **2-OPPh$_3$**.

**Bond Distances and Angles**	**2-OPMe$_2$Ph**	**2-OPMePh$_2$**	**2-OPPh$_3$**
V1-O1	2.0403(19) Å	2.052(14) Å	2.049(3) Å
O=P	1.5032(19) Å	1.487(15) Å	1.506(3) Å
V1-O_c	2.0982(17) Å	2.112(14) Å	2.117(3) Å
V=O_t (avg.)	1.5998 Å	1.6002 Å	1.598 Å
V1-O_b-V_n (avg.)	~105°	~104°	~104°

Table S6. Bond valence sum calculations for the crystallographically independent vanadium ions in **2-OPMe$_2$Ph**, **2-OPMePh$_2$**, and **2-OPPh$_3$** based on X-ray crystallographic data collected at 100 K. Table reflects the results of BVS calculations using V-O bond valence parameters (r_0) for different oxidation states of vanadium.

**2-OPMe$_2$Ph**	**V1**	**V2**	**V3**	**V4**	**V5**	**V6**
V(III)	**3.097**	3.886	4.483	3.9955	3.894	3.950
V(IV)	3.171	**3.979**	4.590	**4.049**	**3.986**	4.044
V(V)	3.409	4.243	**4.886**	4.317	4.250	4.31

**2-OPMePh$_2$**	**V1**	**V2**	**V3**	**V4**	**V5**	**V6**
V(III)	**3.004**	3.909	3.821	3.899	4.049	4.445
V(IV)	3.076	**4.002**	**3.912**	**3.992**	**4.146**	4.551
V(V)	3.308	4.268	4.174	4.257	4.416	**4.845**

**2-OPPh$_3$**	**V1**	**V2**	**V3**	**V4**	**V5**	**V6**
V(III)	**3.097**	4.110	4.500	3.955	3.894	3.953
V(IV)	3.171	**4.208**	4.607	**4.049**	**3.986**	**4.048**
V(V)	3.409	4.487	**4.904**	4.317	4.250	4.315

Table S7. Bond vibrations (cm^{-1}) of **2-OPMe$_3$**, **2-OPMe$_2$Ph**, **2-OPMePh$_2$**, and **2-OPPh$_3$**.

	**2-OPMe$_3$**	**2-OPMe$_2$Ph**	**2-OPMePh$_2$**	**2-OPPh$_3$**
V=O	962 cm^{-1}	962 cm^{-1}	964 cm^{-1}	966 cm^{-1}
O-CH_3	1032 cm^{-1}	1036 cm^{-1}	1036 cm^{-1}	1040 cm^{-1}
P=O	1163 cm^{-1}	1159 cm^{-1}	1159 cm^{-1}	1157 cm^{-1}

Table S8. Relationship between phosphine nucleophilicity (pKa or an electronic parameter derived from ν(CO) of a nickel compound), cone angle, and reaction time for formation of all (OPR_3)-bound clusters.

	**PMe_3**	**PMe_2Ph**	**$PMePh_2$**	**PPh_3**
pKa[4]	8.65	6.50	4.59	2.73
Electronic parameter[5]	2064.1	2065.3	2067.0	2068.9
Tolman Cone Angle[5]	118	122	136	145
Reaction time with **1**	19 hrs	7 hrs	28 hrs	15 days

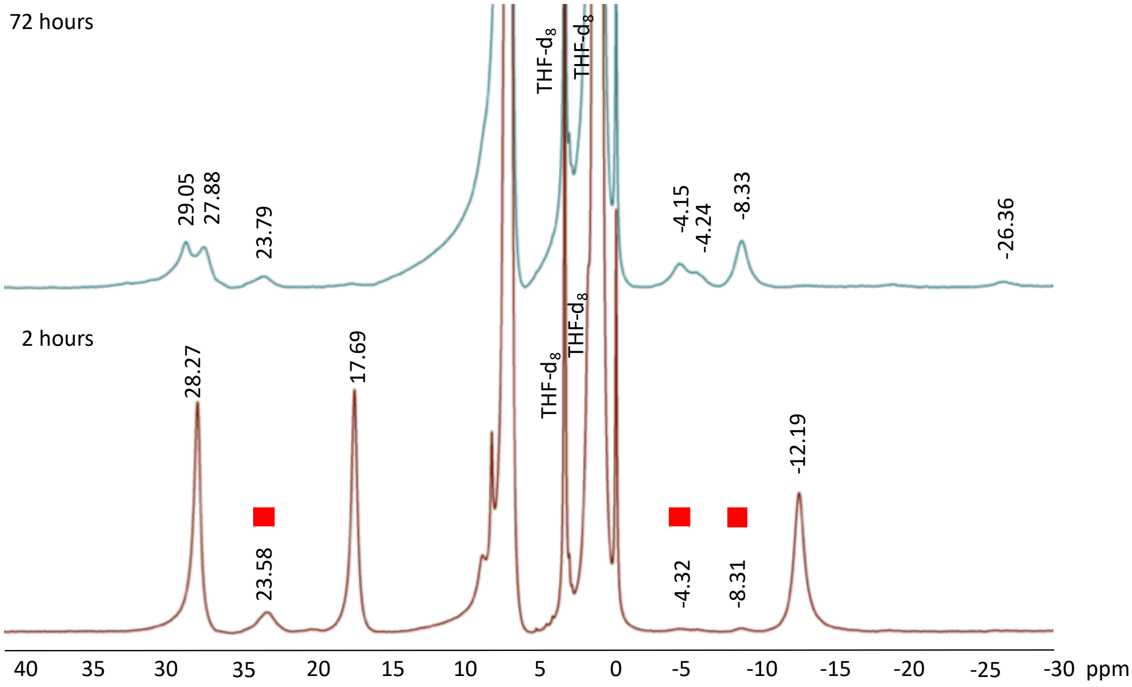

Figure S11. ^{1}H NMR spectrum of a J-young scale reaction of addition of 4 equiv PMe_2Ph to complex **1** in THF-d_8 at 70 ºC. The 2 h time point (bottom, red) shows formation of the mono-vacant product, **2-$OPMe_2Ph$** and the over-reduced molecule, $V_6O_5(OMe)_{12}(OPMe_2Ph)_2$ (denoted by red squares). Allowing the reaction to stir for 72 h (top, blue) results in complete conversion to the ($OPMe_2Ph$)$_2$-bound molecule, confirmed by ESI-MS (Figure S12).

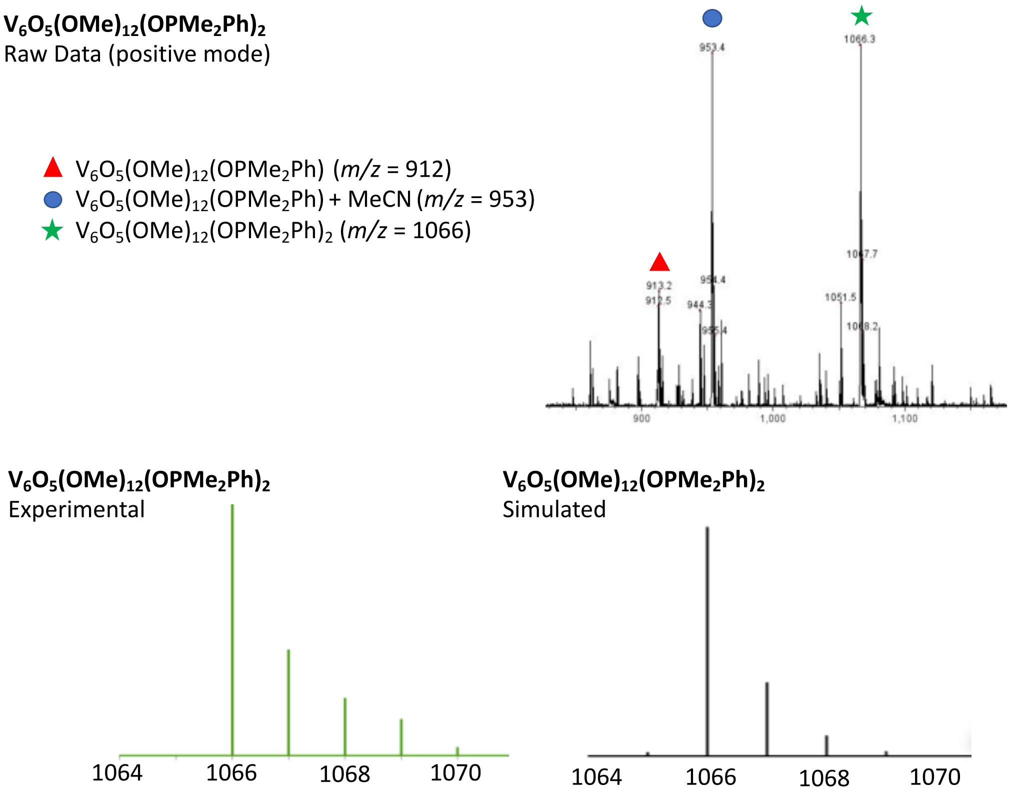

Figure S12. ESI-MS (+)ve of the $(OPMe_2Ph)_2$-bound molecule, $V_6O_5(OMe)_{12}(OPMe_2Ph)_2$ (m/z = 1066).

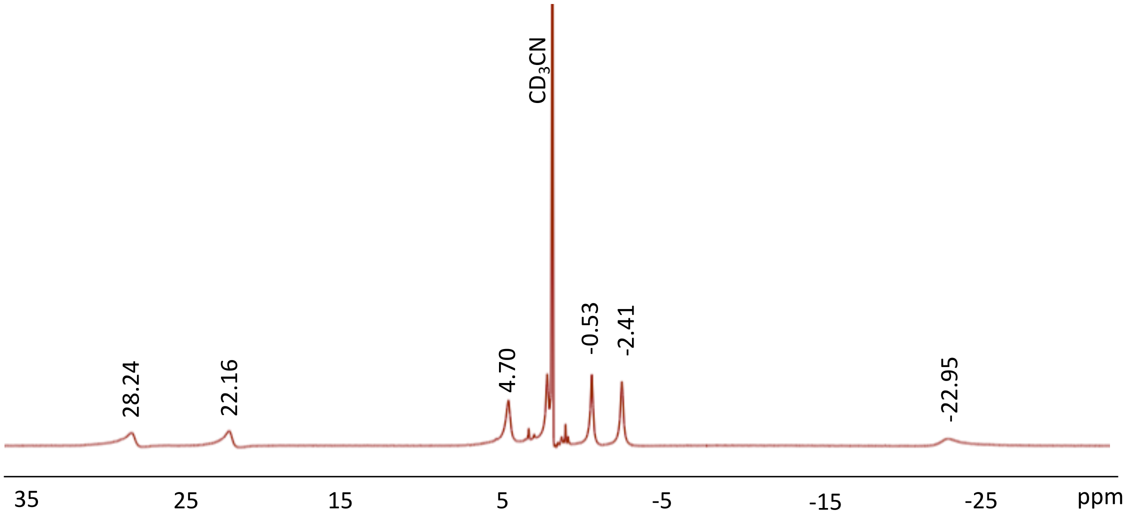

Figure S13. ^{1}H NMR spectrum of **4-OPMe$_3$** in CD_3CN at 21 ºC.

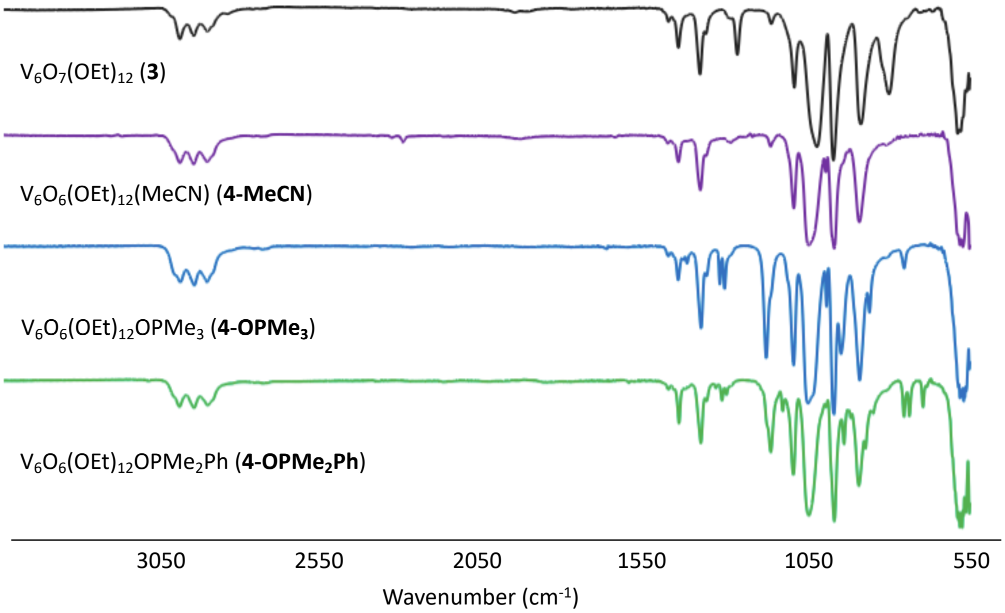

Figure S14. Infrared absorption spectra of **3** (black), **4-MeCN** (purple), **4-OPMe$_3$** (blue), and **4-OPMe$_2$Ph** (green). See Table S9 for bond vibrations.

Table S9. Bond vibrations (cm^{-1}) of **4-MeCN**, **4-OPMe$_3$**, and **4-OPMe$_2$Ph**.

	**4-MeCN**	**4-OPMe$_3$**	**4-OPMe$_2$Ph**
V=O	964 cm^{-1}	964 cm^{-1}	964 cm^{-1}
O-CH_3	1040 cm^{-1}	1042 cm^{-1}	1040 cm^{-1}
P=O	---	1171 cm^{-1}	1157 cm^{-1}

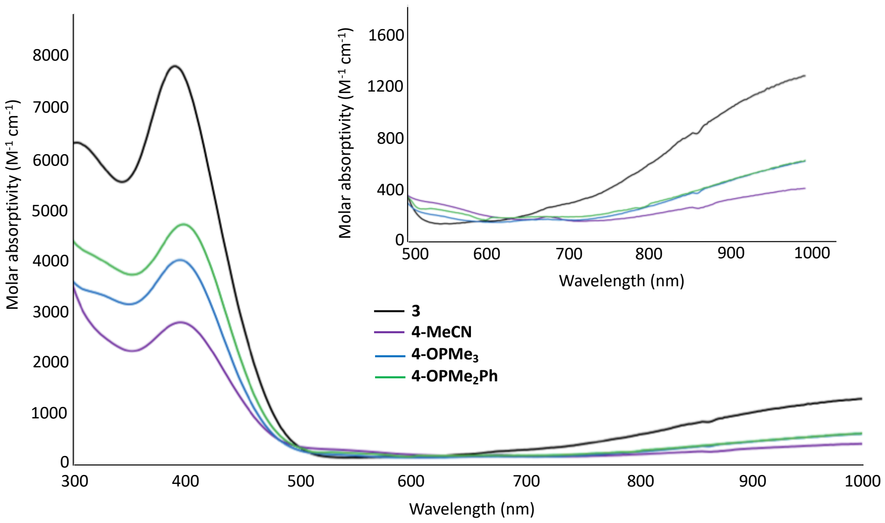

Figure S15. Electronic absorption spectra of complexes **3** (black), **4-MeCN** (purple), **4-OPMe₃** (blue), and **4-OPMe₂Ph** (green) collected in dichloromethane at 21 ºC.

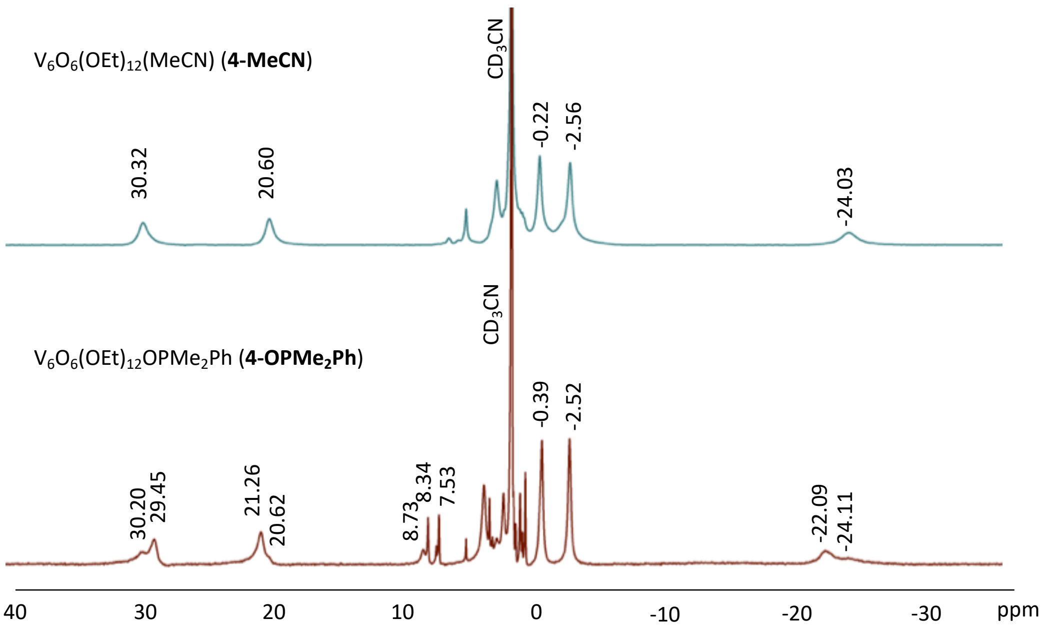

Figure S16. ^{1}H NMR spectra of complexes **4-OPMe₂Ph** (bottom, red), and **4-MeCN** (top, blue) collected in CD_3CN at 21 ºC.

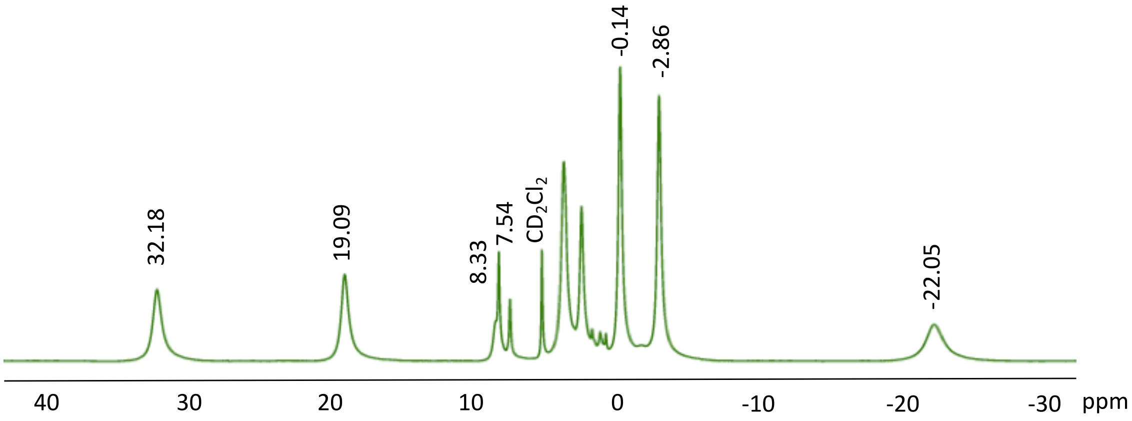

Figure S17. ^{1}H NMR spectra of **4-OPMe$_2$Ph** in CD_2Cl_2 at 21 ºC.

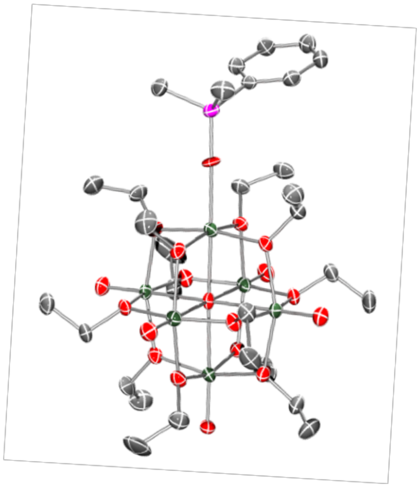

Figure S18. Molecular structure of **4-OPMe$_2$Ph** shown with 50% probability ellipsoids. Hydrogen atoms removed for clarity. The molecules are arranged in head-to-tail chains (the head being the phosphine oxide ligand and the tail being the vanadyl group on the opposite side of the molecule) that are disordered with chains directed in the opposite direction. Due to the disorder and the restraints required to model it, bond lengths and angles cannot be obtained.

Table S10. Crystallographic Parameters of **4-OPMe$_2$Ph** (CCDC: 1914250).

Empirical formula	$C_{32}H_{71}PO_{19}V_6$	
Formula weight	1096.49	
Temperature	100.0(3) K	
Wavelength	1.54184 Å	
Crystal system	Monoclinic	
Space group	*Cc*	
Unit cell dimensions	a = 20.7441(3) Å<br>b = 10.74980(16) Å<br>c = 21.6524(3) Å	$\alpha = 90^o$<br>$\beta = 101.7536(14)^o$<br>$\gamma = 90^o$
Volume	4727.14(12) Å^3	
Z	4	
Reflections collected	45966	
Independent reflections	9141	
Goodness-of-fit on F2	1.055	
Final R indices [I>2sigma(I)]	R1 = 0.0568, wR2 = 0.1553	

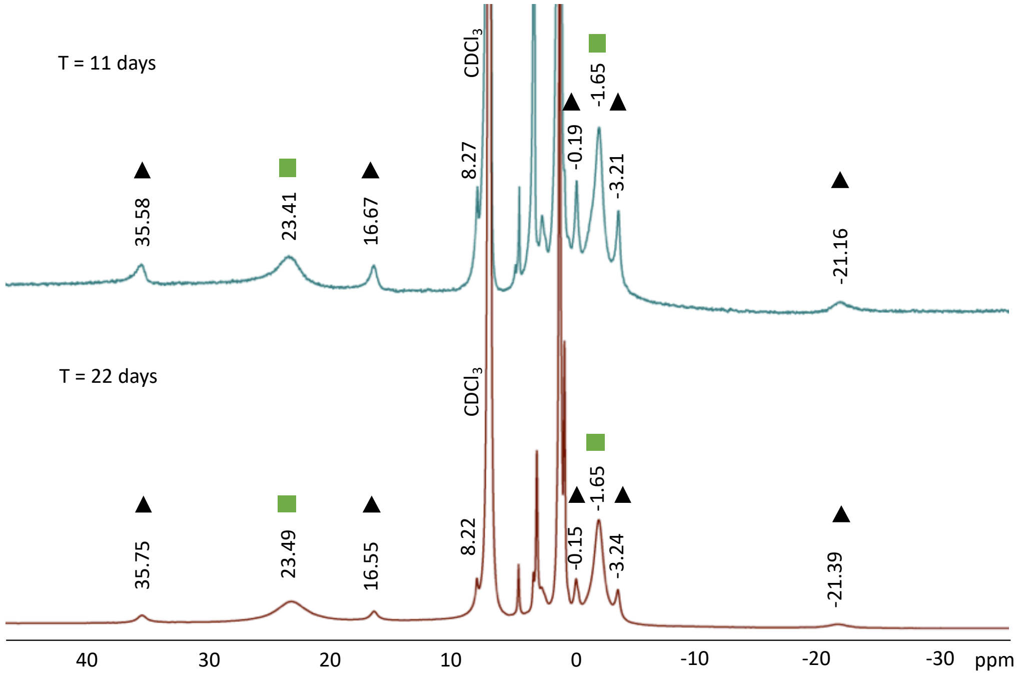

Figure S19. ^{1}H NMR spectra of attempted synthesis of **4-OPMePh₂** (black triangles) collected in $CDCl_3$ at 21 ºC. Time points following addition of $PMePh_2$ (4 equiv) to **3** (green squares) after stirring at 70 ºC for over two weeks in a 15 mL pressure vessel show incomplete conversion to **3**.

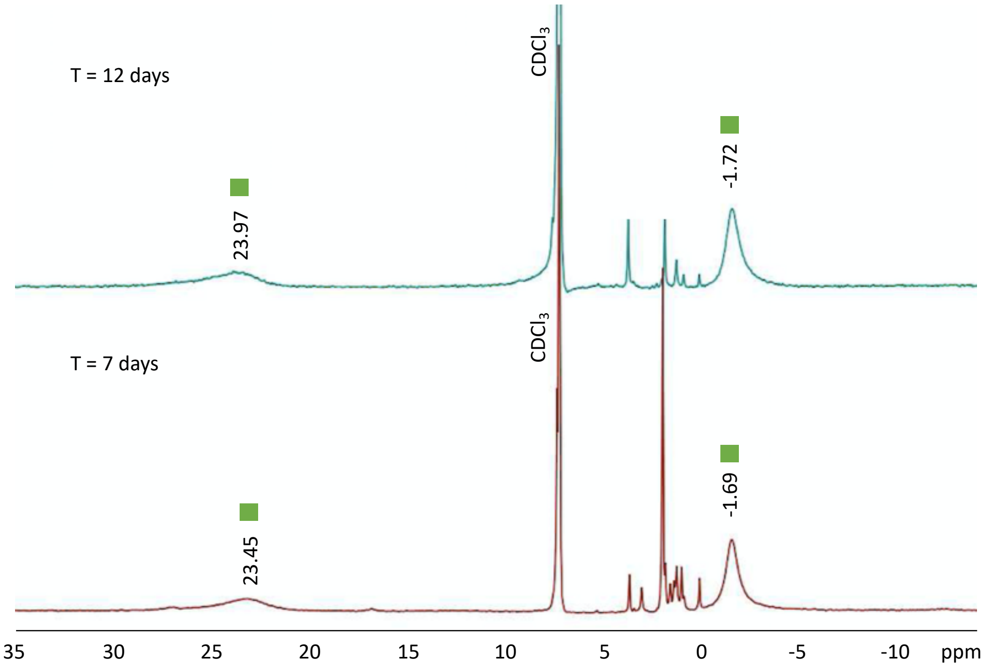

Figure S20. ^{1}H NMR spectra of attempted synthesis of **4-OPPh₃** collected in $CDCl_3$ at 21 ºC. Time points following addition of PPh_3 (4 equiv) to **3** after stirring at 70 ºC for over a week in a 15 mL pressure vessel show no conversion of **3** (green squares) after 12 days.

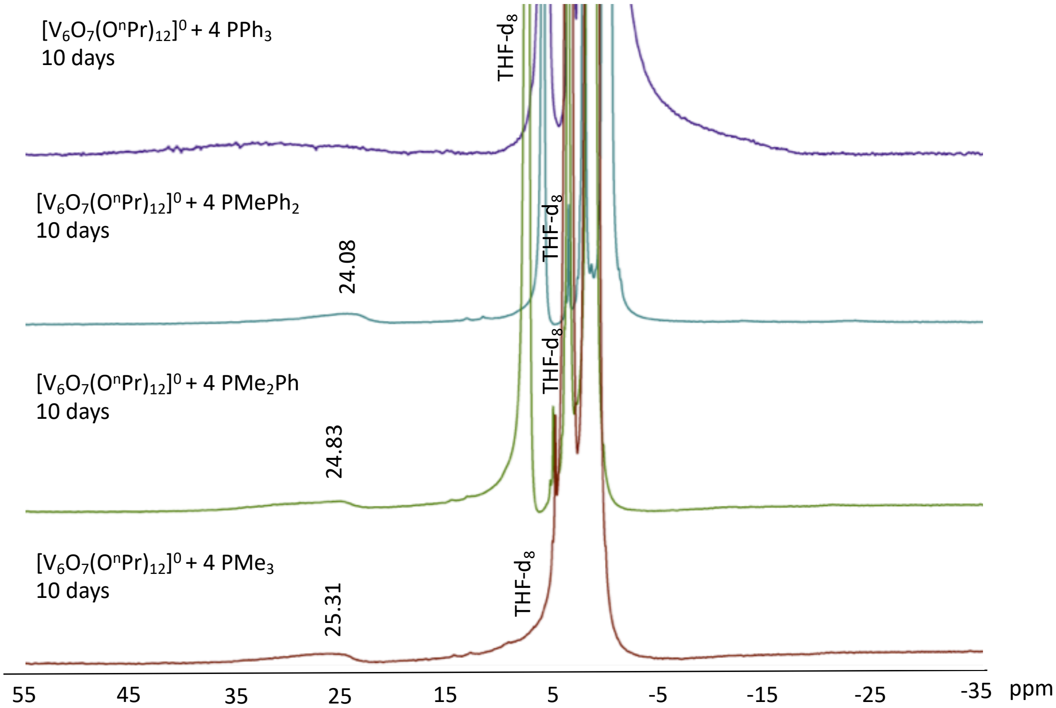

Figure S21. ^{1}H NMR spectra of a mixture of the propoxide-bridged cluster, $[V_6O_7(O^nPr)_{12}]^0$, and a series of phosphanes (4 equiv). Spectra were collected in THF-d_8 at 21 ºC. All mixtures were heated to 70 ºC for 10 days in a J-young tube. Time points show no conversion of $[V_6O_7(O^nPr)_{12}]^0$.

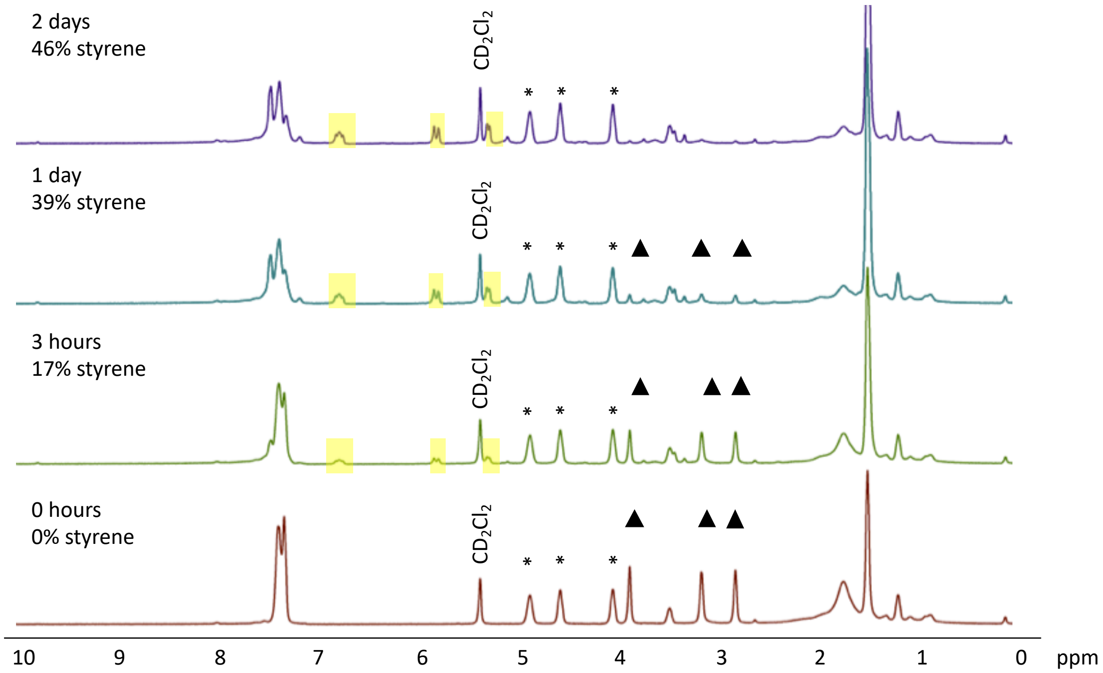

Figure S22. Representative ^{1}H NMR spectra of the diamagnetic region of a crude reaction mixture of **2-OPMe₃** and styrene oxide (1 equiv) at 70 ºC in CD_2Cl_2. Time points reveal loss of styrene oxide (black triangles) and growth of styrene (yellow highlight). Propylene carbonate (denoted by '*') was used as a ^{1}H NMR internal standard.

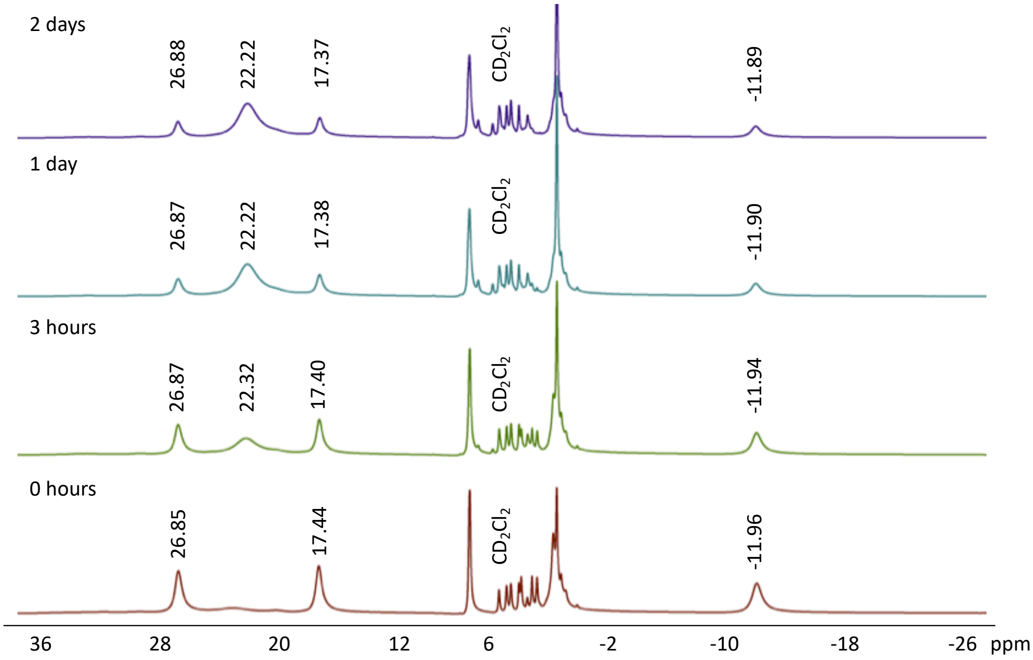

Figure S23. ^{1}H NMR spectra highlighting the paramagnetic region of the crude reaction mixture of **2-OPMe$_3$** and styrene oxide (1 equiv) at 70 ºC in CD_2Cl_2. Time points show growth of a resonance corresponding to complex **1** (22.2 ppm).

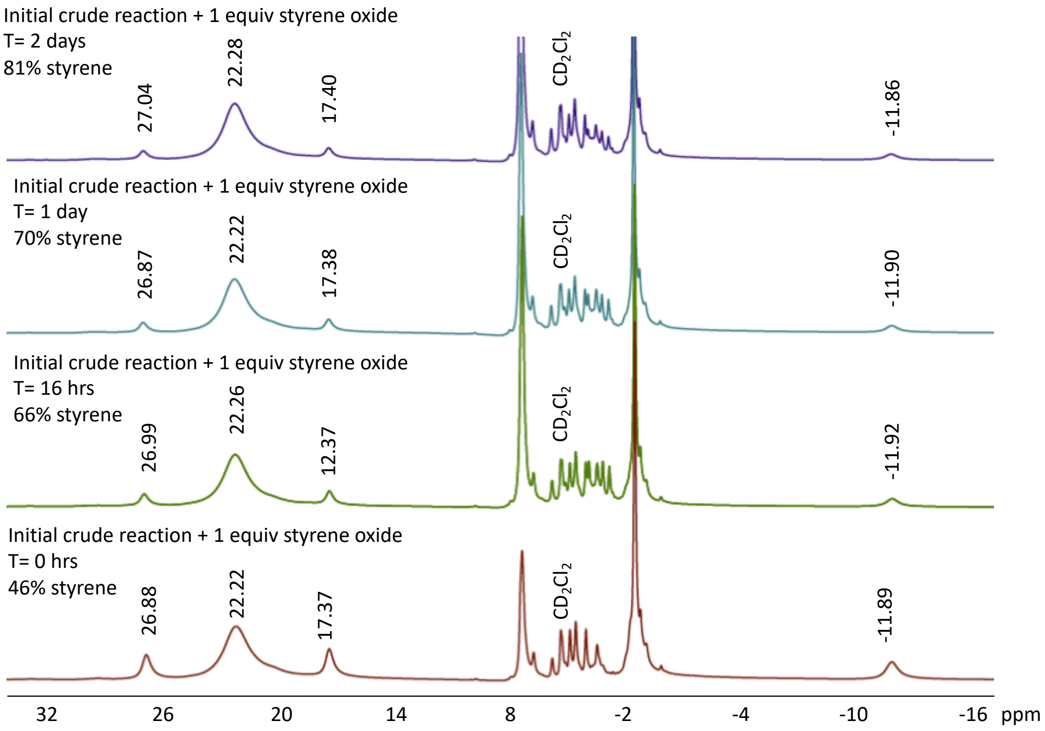

Figure S24. ^{1}H NMR spectra of the addition of *excess* styrene oxide (1 equiv) to the crude reaction mixture of **2-OPMe$_3$** and styrene oxide after 2 days at 70 ºC (CD_2Cl_2, see Figure S23 for initial time points of this reaction). Time points show further oxidation of **2-OPMe$_3$** to **3** (23.4, -1.60 ppm) after an additional 2 days. Yields reported are total yields from both experiments.

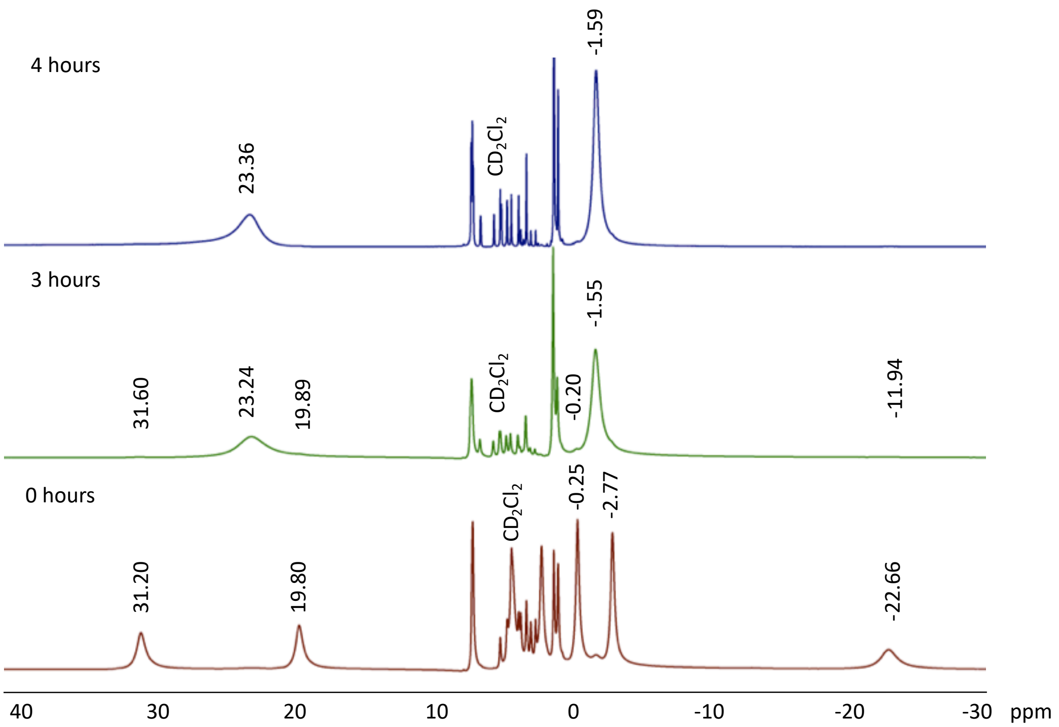

Figure S25. ^{1}H NMR spectra of paramagnetic region of the crude reaction mixture of **4-OPMe$_3$** and styrene oxide (1 equiv) at 70 ºC in CD_2Cl_2. Time points show complete conversion of **4-OPMe$_3$** to **3** (23.4, -1.60 ppm) after 4 hours.

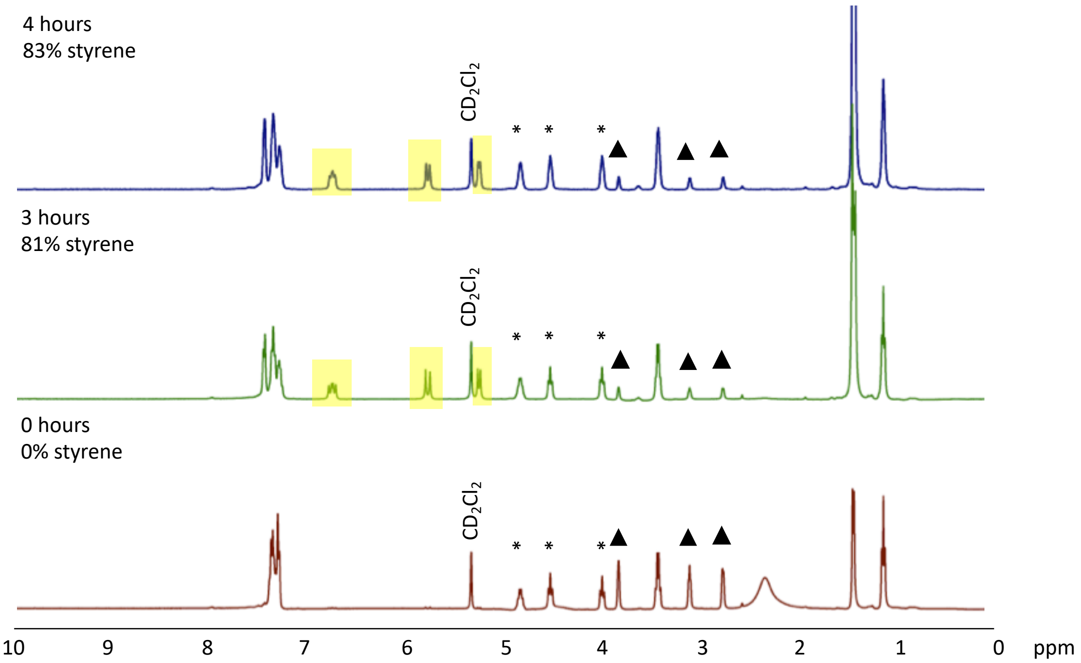

Figure S26. ^{1}H NMR spectra of the diamagnetic region of a crude reaction mixture of **4-OPMe$_3$** and styrene oxide (1 equiv) at 70 ºC (CD_2Cl_2). Time points reveal loss of styrene oxide (black triangles) and growth of styrene (yellow highlight). Propylene carbonate (denoted by '*') was used as a ^{1}H NMR internal standard.

References:

1. C. Daniel and H. Hartl, *J. Am. Chem. Soc.*, 2005, **127,** 13978-13987.
2. B. E. Petel, W. W. Brennessel and E. M. Matson, *J. Am. Chem. Soc.*, 2018, **140,** 8424-8428.
3. B. E. Petel, A. A. Fertig, M. L. Maiola, W. W. Brennessel and E. M. Matson, *Inorg. Chem.*, 2019, *Advanced Article*, DOI: 10.1021/acs.inorgchem.9b00389.
4. M. N. Golovin, M. M. Rahman, J. E. Belmonte and W. P. Giering, *Organomet.*, 1985, **4**, 1981-1991.
5. C. A. Tolman, *Chem. Rev.*, 1977, **77**, 313-348.
